# Targeting the STING pathway in tumor-associated macrophages regulates innate immune sensing of gastric cancer cells

**DOI:** 10.7150/thno.37745

**Published:** 2020-01-01

**Authors:** Lei Miao, Jingjing Qi, Qi Zhao, Qi-Nian Wu, Da-Liang Wei, Xiao-Li Wei, Jia Liu, Jun Chen, Zhao-Lei Zeng, Huai-Qiang Ju, Hui-yan Luo, Rui-Hua Xu

**Affiliations:** 1 State Key Laboratory of Oncology in South China, Sun Yat-sen University Cancer Center, Collaborative Innovation Center for Cancer Medicine, Guangzhou 510060, China.; 2 Department of Pediatric Surgery, Guangzhou Women and Children's Medical Center, Guangzhou Medical University, Guangzhou, Guangdong, China.; 3 Department of Pathology, Sun Yat-sen University Cancer Center, State Key Laboratory of Oncology in South China, Collaborative Innovation Center for Cancer Medicine, Guangzhou 510060, China.; 4 Department of Medical Oncology, Sun Yat-sen University Cancer Center, State Key Laboratory of Oncology in South China, Collaborative Innovation Center for Cancer Medicine, Guangzhou 510060, China.; 5 Zhongshan School of Medicine, Sun Yat-sen University, Guangzhou 510060, China.

**Keywords:** Gastric carcinoma, STING, apoptosis, IL-24, IL-6R

## Abstract

***Rationale:***STING is a critical player in the innate and adaptive immune system, sensing cytosolic DNA to activate the expression of interferon genes and regulate T lymphocytes, which drives immunogenic responses to cancer cells. Tumor-associated macrophages (TAMs), abundantly present in the tumor microenvironment, play a key role in cancer development. Gastric cancer is one of the most common and leading causes in cancer-related death worldwide. However, studies on the function and regulation of STING in TAMs and their roles in gastric cancer progression are still limited.

***Methods:*** We analyzed STING and CD68 expression of 200 pairs of gastric cancer and adjacent normal tissues by immunohistochemistry to identify the prognostic values of STING, as well as the correlations between STING and CD68 in gastric cancer. The characteristics of STING-altered macrophages, as well as their effects on cancer cell apoptosis and T cell differentiation were examined by flow cytometry. Cytokines secreted by STING-altered macrophages were identified by the Human Inflammation Array3 kit. Concentrations of soluble IL24 and IFN-β were measured by ELISA. *In vivo* models, including spontaneous gastric cancer in p53^+/-^ mice and cell line-based xenografts, were established, and clinical benefits of STING-altered macrophages were examined.

***Results:*** Our study identifies STING as a prognostic factor for gastric cancer, and for the first time demonstrated that knocking-down STING and STING activation by 2'3'-c-GAMP both promote TAMs polarizing into pro-inflammatory subtype and induce apoptosis of gastric cancer cells, mechanistically through IL6R-JAK-IL24 pathway.

***Conclusions***: This study evaluated effects of targeting STING in TAMs in anti-gastric-cancer therapies. Moreover, we unveil a novel function of STING to activate the IL6R-JAK-IL24 pathway in macrophages.

## Introduction

Immune cells induce anti-tumor response after exposing to antigens of accumulated mutations in cancer cells 1. As the first line of defense, the innate immune system is important to initiate adaptive immunity to cancer cells 2. Innate immunity can identify “self” and “non-self” through various pattern recognition receptors (PRRs) such as DNA or RNA sensors in the cytosol 3. As a critical early event against viral infection, type I interferon (IFN) is induced and activates the transcription of IFN-stimulated genes that establish an antiviral innate immune state to limit the spread of viruses. Crucial to the induction of type I IFN is the recognition of viral pathogen-associated molecular patterns by pattern recognition receptors, among which, the cyclic GMP-AMP synthase (cGAS)-stimulator of interferon genes (STING) modulates the antiviral response triggered by DNA viruses and retroviruses. STING is a cytoplasmic DNA sensor, anchored in the endoplasmic reticulum 4-6. After activation, STING translocates to the periphery of the nucleus and recruits TBK1 to activate IRF3, thereby inducing expression of interferon and production of specific chemokines 7. Cancer cells can down-regulate STING activity to resist immune cell-induced apoptosis in various mouse models 8-10. STING downregulation also dampens the immunogenicity of neck squamous cell carcinoma (HNSCC), mainly manifested by the reduction of tumor-infiltrating CD3^+^CD8^+^ T cells and the decrease of type I interferon and immune cells-recruiting chemokines 11. Moreover, activation of the STING pathway by small-molecule activators in immune cells promotes the anti-tumor inflammatory response in mice 12.

As one of the most common cancers and leading causes in cancer-related death, gastric cancer (GC) is an important health problem in China, Japan and Korea 13, 14. For the last decade, clinical practice has managed to enhance adaptive anti-tumor immunity by immunotherapies, such as oncolytic viruses, chimeric antigen receptor T cells (CAR-Ts), bispecific antibodies, and immune checkpoint inhibitors 15-19. Due to the significant heterogeneity of GC revealed by genomic profiling, the GC-associated immune microenvironment and prognosis factors of immunotherapies remain poorly understood. As important innate immune cells in the tumor microenvironment, tumor-associated macrophages (TAMs) play a key role in tumor development and immunotherapy. High densities of TAMs associate closely with poor survival of GC patients 20, 21. TAMs often undergo phenotype polarization in response to stimuli or inhibitory factors, either to pro-inflammatory or anti-inflammatory subtypes, which cause immune response or immune escape of the tumors, respectively 1, 22, 23. Clinical studies have confirmed that anti-inflammatory TAMs are negatively correlated with patients' prognosis 24-26. Moreover, the infiltration and polarization of TAMs can be used as an independent prognosis factor of GC, and combined with TNM staging, makes a more effective and dependable prediction of the prognosis of patients 27. Besides, the TAMs spread in the peritoneum of GC patients are normally polarized to anti-inflammatory subtype, which can promote the growth and progression of GC 28. In contrast to anti-inflammatory, pro-cancer subtype, pro-inflammatory macrophages can kill tumor cells through inducing hemorrhagic necrosis of the tumor blood vessels, or secreting chemokines to recruit effector T cells, thereby exerting anti-tumor effects 22. These macrophages can also maintain high expression of the enzyme inducible nitric oxide synthase (iNOS) and other cytotoxic molecules 29. Pro-inflammatory macrophages also directly induce apoptosis of fibro/adipogenic progenitors through their expression of tumor necrosis factor (TNF) 30, which in turn triggers the induction of a RIPK1-FADD-caspase8 apoptotic complex 31-34. Moreover, inhibition of the polarization of pro-inflammatory macrophages can accelerate the development of precancerous lesions in GC 20. Therefore, targeting TAMs, especially polarizing TAMs to pro-inflammatory phenotype, may become a novel strategy of tumor immunotherapy in the future.

Given the essential roles played by STING in innate immunity and significant effects of TAMs on GC progression, we set up the study to specifically explore the role of STING and its downstream pathway in macrophages in GC progression. Our results identified STING as a prognostic factor for GC, and for the first time showed that knocking-down STING and STING activation by 2'3'-c-GAMP both promote TAMs differentiating into pro-inflammatory subtype and induce apoptosis of gastric cancer cells by activating IL6R-JAK-IL24 pathway. *In vivo* studies further showed that knocking-down STING or STING activation have therapeutic effects on endogenous gastric cancer or xenografted tumors, and can promote the effectiveness of T cells. Taken together, this study provides evidence for targeting macrophages in anticancer therapies and unveils a novel function of STING on macrophages' polarization and T cell activation.

## Materials and Methods

### Tissue specimens, immunohistochemistry (IHC) and immunofluorescence

The study was approved by the Institutional Ethics Committee of Sun Yat-sen University Cancer Center (SYSUCC). 200 pairs of formalin-fixed, paraffin-embedded GC samples and normal adjacent tissues (>2 cm from tumor), along with the available clinicopathological information, were obtained from SYSUCC with informed consents.

IHC staining was performed as previously described 35. The sample slides from patients and mice were de-waxed, rehydrated, antigen-retrieved, permeabilized, and blocked before hybridization with rabbit anti-STING antibody (Cat# 13674, Cell Signaling Technology, 1:500), mouse anti-human CD68 antibody (Cat# ab955, Abcam, 1:200), rabbit anti-Ki67 antibody (D3B5) (Cat#12202, Cell Signaling Technology, 1:500), rabbit anti-CD8α antibody (D4W2Z) (Cat# 98941, Cell Signaling Technology, 1:500) at 4 °C overnight, followed by incubation with biotinylated goat anti-rabbit/mouse immunoglobulin (GK500705, DAKO) at 37 °C for 30 min. Finally, the slides were visualized using diaminobenzidine (DAB) Reagents (GK500705, DAKO). Five representative fields from each section were assessed by two experienced pathologists. For IHC grading, the scores of positive staining in each field were defined as percentage of staining in the whole section, and the staining intensity is defined as no (0), weak (1), medium (2), and strong (3). The immunoscore was generated by multiplying these two scores.

For immunofluorescence analysis, the tissue slides and cells were incubated with rabbit anti-STING antibody (Cat# 13674, Cell Signaling Technology, 1:500), mouse anti-human CD68 antibody (Cat# ab955, Abcam, 1:200), as well as rabbit anti-IL24 antibody (Cat# orb184288, biorbyt, 1:500), followed by incubation with anti-mouse/rabbit Alexa Fluor secondary antibodies (Cat# 4410, Cat# 4412, Cell Signaling Technology, 1:1000). DAPI was used for nuclear staining. The images were captured using an Olympus FluoView1000 laser scanning confocal microscope (Olympus Corporation) equipped with a ×40 objective.

### Reagents, cell culture, and treatments

The JAK inhibitor Tofacitinib (Cat# 14703) was from Cell Signaling technology; 2'3'-c-GAMP (Cat# tlrl-nacga) was from invivogen; Phorbol 12-myristate 13-acetate (PMA, Cat# P8139) was from Sigma-Aldrich. The human GC cell line HGC27 and mouse GC cell line MFC were obtained from the American Type Culture Collection (ATCC) and cultured according to instructions. THP1-Dual^TM^ KO-STING (Cat# thpd-kostg) and THP1-Dual^TM^ cells (Cat# thpd-nfis) were from invivogen. Cell lines were all authenticated based on STR fingerprinting by the Forensic Medicine Department of Sun Yat-sen University (Guangzhou, China).

Fresh gastric tumor samples were minced into small pieces and digested in collagenase I (Gibco) and trypsin (Gibco) (V:V = 1:15) at 37 °C for 1 h. The cells were subsequently filtered through a 40 μm cell strainer and centrifuged at 200×g for 5 min, then washed with PBS for 2 times, resuspended and cultured in RPMI-1640 medium containing 10% FBS and 1% penicillin-streptomycin.

Peripheral mononuclear cells (PBMC) were isolated from the blood of healthy donors by Ficoll density gradient centrifugation (Cat# 45-001-749, GE Healthcare). Monocytes were isolated by positive selection using anti-CD14 microbeads (Cat# 130-050-201, Miltenyi Biotec). The CD14^+^ monocytes were cultured in RPMI-1640 medium containing 10% FBS supplemented with 20 ng/mL M-CSF (Cat# 30025, PeproTech) for 7-10 days to differentiate into mature macrophages.

Tumor-specific CD3^+^ T cells were purified by a negative-selection procedure using a Pan T Cell Isolation Kit (Cat# 130-096-535, Miltenyi Biotec). CD3^+^ T cells were cultured in serum-free ImmunoCult-XF T Cell Exp Medium (StemCell) containing ImmunoCult™ Human CD3/CD28 T Cell Activator (Cat# 10971, StemCell) and human IL-2 (Cat# 200-02, PeproTech) for 5 days, then stained with PE anti-human CD25 antibody (Cat# 302606, BioLegend) for flow cytometry analysis to detect T cell activation.

### Western blot

Cells were incubated and lysed with ice-cold RIPA buffer containing complete protease inhibitors and phosphatase inhibitor cocktail. Protein concentrations were quantified with a BCA protein assay kit (Thermo Scientific). All samples were diluted into equal protein concentration. Western blot analysis was performed as previously described 36. Primary antibodies included anti-STING (Cat# 13674, Cell Signaling Technology, 1:1000), anti-CD68 (Cat# ab955, Abcam, 1:1000), anti-GAPDH (Cat# ab181602, Abcam, 1:10000), anti-phospho-STAT1 (Cat# 9167, Cell Signaling Technology, 1:1000), anti-STAT1 (Cat# 14994, Cell Signaling Technology, 1:1000), anti-phospho-STAT3 (Cat# 9145, Cell Signaling Technology, 1:1000), anti-STAT3 (Cat# 12640, Cell Signaling Technology, 1:1000), anti-Vinculin (Cat# ab129002, Abcam, 1:10000), anti-β-actin (Cat# 3700, Cell Signaling Technology), and anti-α-tubulin (Cat# ab7291, Abcam, 1:5000). The bands were visualized by enhanced chemiluminescence (Millipore).

### Cell transfection, adenovirus transduction and CRISPR-Cas9 knockout

The full-length cDNAs of human STING and mouse Sting were cloned and assembled into the vector pAdeno-MCMV-MCS-EGFP-3FLAG. The shRNA sequences were synthesized and assembled into the vector pDKD-CMV-Puro-U6-shRNA. Constructs were verified and packaged into adenoviruses by OBiO Technology Inc.(Shanghai, China). Adenoviruses were further used to transduce and select macrophages. The shRNAs targeting human STING are #1: CCATGTCACAGGATGCCAA, #2: CCCACAGACGGAAACA GTT; targeting mouse Sting are #1: GCATTACAACAACCTGCTA, #2: GCCAGCGGCTGTATATTCT; and targeting human IL24 are #1: GCAAAGCCTGTGGACTTTA, #2: CCAACAACTTTGTTCTCAT. The scrambled shRNA sequence (SC): CCGGTTCTCGAACGTGTCACGTTTCAAGAGAACGTG ACACGTTCGGAGAA TTTTTTG gene knockdown and overexpression, respectively. The “control” in our studies stands for the average of undistinguishable controls of scrambled sequence, empty vector, and PBS treatment. As previously reported 37, two Sting guide RNAs (gRNA1: CACCTAGCCTCGCACGAACT; gRNA2: TATTTGGAGCGGTGACCTCT) were used to generate complete Sting knockout clones in mouse BM-DMs (OBiO Technology Inc, Shanghai), a control transfected with scrambled sequence gRNA was used in the study.

### RNA extraction and Real-time PCR

Total RNA was extracted from macrophages and tissues using TRIzol reagent (Life Technologies) and converted to cDNA according to the manufacturer's instructions. Real-time PCR was performed using the PrimeScript RT Master Mix (Cat# RR036A, TAKARA) 36. The expression of the target gene was normalized to GAPDH, and the fold change was calculated as 2^-ΔΔCT^ method. Specific primer sequences are listed in Supplementary Table 1.

### Flow cytometry

The TAMs separated from fresh gastric tumor samples, as well as human or mouse macrophages were harvested and washed twice with PBS, then stained with PerCP-Cy™5.5 anti-Human CD45 antibody (Cat#564106, BD Bioscience), FITC anti-human CD11b antibody (Cat#301329, BioLegend), PE anti-human CD163 antibody (Cat#326505, BioLegend), APC anti-mouse CD80 antibody (Cat# 104714, BioLegend), and FITC anti-mouse CD206 (MMR) antibody (Cat# 141703, BioLegend) for flow cytometry analysis.

Tumor-specific T cells cocultured with human macrophages were stained with FITC anti-human CD8 antibody (Cat# 344704, BioLegend), PE/Dazzle™ 594 anti-human CD4 antibody (Cat# 357412, BioLegend), and PE/Cy7 anti-human CD3 antibody (Cat # 300316, BioLegend) for human T cell differentiation analysis. T cells from mouse blood and spleen samples were harvested and filtered, followed by staining with APC/Cy7 anti-mouse CD3ε antibody (Cat# 100329, BioLegend), PerCP/Cy5.5 anti-mouse CD4 antibody (Cat# 100433, BioLegend), PE/Dazzle™ 594 anti-mouse CD8a antibody (Cat# 100761, BioLegend) for mouse T cell differentiation analysis.

PerCP-Cy™5.5 Mouse IgG1 κ Isotype Control (Cat#550795, BD Bioscience), FITC Mouse IgG1, κ Isotype Ctrl Antibody (Cat#400107, BioLegend), PE Mouse IgG1, κ Isotype Ctrl Antibody (Cat#981804, BioLegend), APC Mouse IgG1, κ Isotype Ctrl Antibody (Cat#400119, BioLegend), PE Rat IgG2b, κ Isotype Ctrl Antibody (Cat#400607, BioLegend), APC Armenian Hamster IgG Isotype Ctrl Antibody (Cat#400911, BioLegend), FITC Rat IgG2a, κ Isotype Ctrl Antibody (Cat#400505, BioLegend), PE/Dazzle™ 594 Rat IgG2b, κ Isotype Ctrl Antibody (Cat#400659, BioLegend), PE/Cy7 anti-human CD3 Antibody (Cat#300316, BioLegend) and APC/Cy7 Armenian Hamster IgG Isotype Ctrl Antibody (Cat#400927, BioLegend), were all identified as isotype controls of tumor tissues and macrophages (human and mouse) for flow cytometric staining.

For cell apoptosis analysis, human or mouse macrophages were seeded in the top chambers of a 6-well transwell plate (Cat#353090, Corning), while human HGC27 or mouse MFC cells were seeded in the bottom chambers (5:1). Cancer cells were harvested and washed twice with PBS after 48 h, resuspended in 500 μL of staining buffer plus propidium iodide (PI) and Annexin-V-FITC. The percentages of cells undergoing apoptosis were evaluated by flow cytometry.

### Enzyme-linked immunosorbent assay (ELISA)

Human and mouse macrophages were seeded and treated for 48 h. Supernatants were collected after centrifugation, IL-24 was measured by mouse Interleukin-24 (Cat# CSB-EL011640MO, cusabi) and human IL-24/MDA-7 ELISA Kit (Cat# CHE0085, 4A biotech), respectively; IFN-β was measured by mouse IFN-β (Cat# CSB-E04945m, cusabi) and human IFN-β ELISA Kit (Cat# CSB-E09889h, cusabi), respectively.

### Reporter assay

Both THP1-Dual^TM^ and THP1-Dual^TM^ KO-STING cells stably express inducible luciferase reporter gene, which is under the control of an ISG54 (interferon-stimulated gene) minimal promoter in conjunction with five IFN-stimulated response elements. The expression of IFN-β was identified by assessing the activity of luciferase, according to the manufacturer's instructions.

### Cell proliferation assay

For the cell viability assay, 1.0×10^5^ HGC-27 and MFC cells were seeded per well in a 6-well plate and incubated overnight, and then cocultured with indicated groups of macrophages. After 72 h, HGC-27 and MFC cells were fixed in methanol and stained with 0.2% crystal violet to manifest their clones.

### *In vivo* treatment protocols

C57BL/6J p53^+/-^ mice were purchased from Biomodel (Shanghai). Spontaneous GC was induced by feeding mice with N-nitroso-sarcosine-ethyl (NSEE) for 16 weeks. The mice were then depleted of macrophages by administration of clodronate liposomes (CL2MDP, Clodronate Liposomes.org) via intraperitoneal injection (1 mg per mouse); PBS was also administrated as a negative control. All macrophage-depleted mice were then randomly assigned into 5 groups, injected intraperitoneally with PBS (n = 10), control bone marrow-derived macrophages (control BM-DMs, n = 10), shSting BM-DMs (n = 10), 2'3'-c-GAMP-treated BM-DMs (n = 10), and Sting-overexpressing BM-DMs (n = 10), respectively. To visualize the exogenous BM-DMs, BM-DMs were incubated with pHrodo™ Red BioParticles™ Conjugate (Cat# P35361, Invitrogen). Red fluorescence imaging of mouse stomachs was performed using the In-Vivo BRUKER FX PRO.

When the subcutaneous MFC tumors were palpable, the mice were randomly divided into 5 groups (6 mice per group): the PBS, control BM-DMs, shSting BM-DMs, 2'3'-c-GAMP-treated BM-DMs, and Sting-overexpressing BM-DMs were injected intraperitoneally, respectively. Tumor sizes were calculated by the formula: volume = width^2^ x length/2, and measures were recorded every 3 days. All animal studies were approved by the Institutional Animal Care and Use Committee at SYSUCC.

### Cytokine measurement and verification

Cytokines secreted by STING-altered macrophages and controls were detected and quantified by Quantibody® Human Inflammation Array 3 (Cat# QAH-INF-G3-4, Raybiotech), according to manufacturer's instructions. Functions and pathway enrichment of differentially expressed cytokines were analyzed by Gene Ontology (GO) and KEGG pathway databases. Differentially expressed cytokines are listed in Supplementary Table 2.

### Statistical Analysis

All the Western blots shown were representative results from at least two independent biological replicates. All the statistical analyses were derived from multiple independent experiments, which were repeated at least twice. Bar graphs represent the mean ± SD and the comparisons were analyzed by Student's *t*-test (unpaired, two-tailed). Kaplan-Meier analysis and the log-rank test were performed to compare survival between two groups of patients. All statistical calculations were performed using GraphPad Prism 5.0 software (La Jolla, CA, USA). p < 0.05 was considered statistically significant.

## Results

### High expression of STING in TAMs predicts poor survival of gastric cancer patients

**Figure 1:**
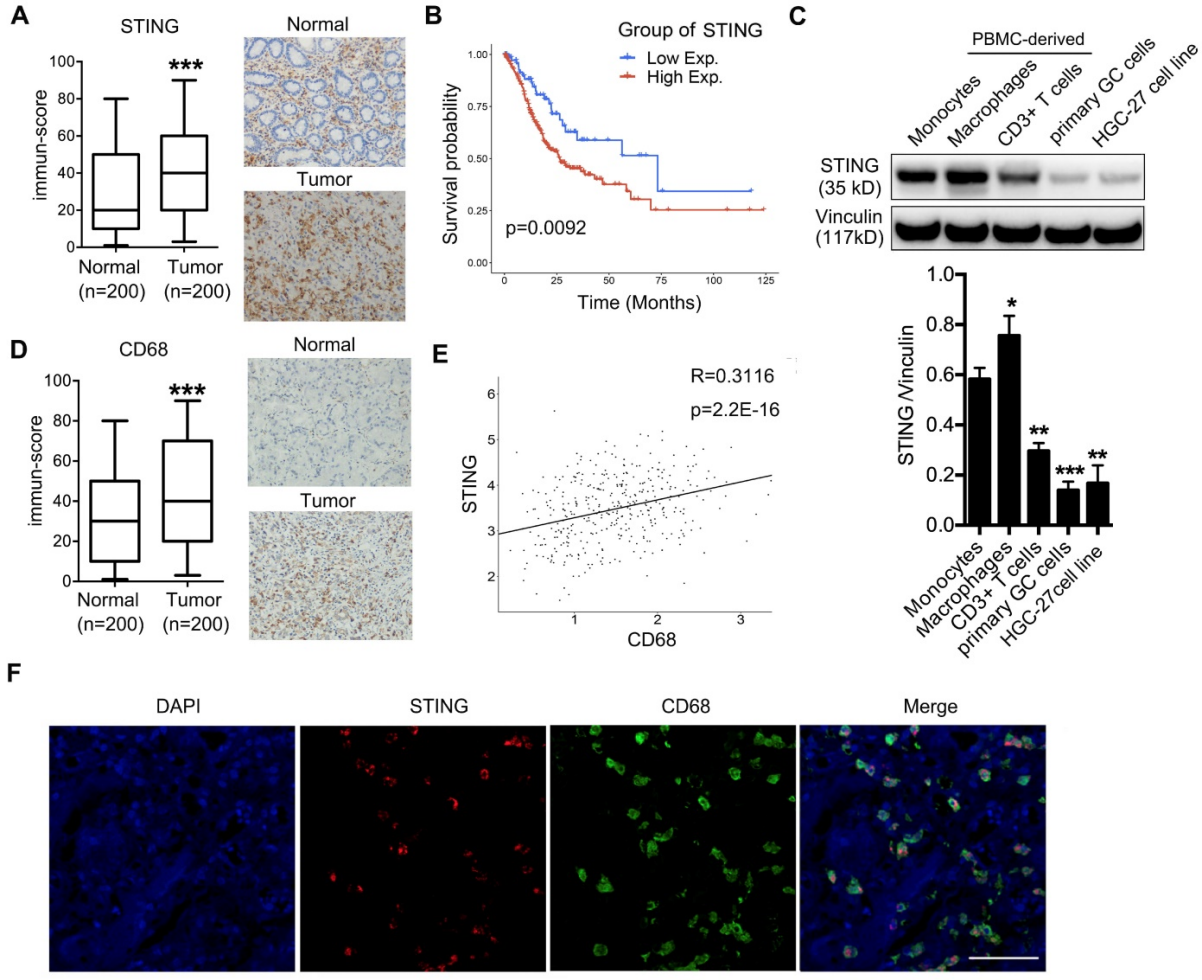
** High STING expression is enriched in macrophages, predicting poor survival of gastric cancer patients. (A)** Left panel, immune-score of STING expression in normal mucosa and gastric tumors; right panel, representative pictures of STING IHC staining in adjacent normal mucosa and tumor tissue of a GC patient. ***, p < 0.001.** (B)** Kaplan-Meier analysis showing overall survival of GC patients with high vs. low STING expression. The STING intensity that can best separate the low and high groups is used as the cut-off.** (C)** Upper panel, immunoblot analysis showing STING expression in indicated cells. Vinculin was used as a loading control. Lower panel, the ratio of STING/Vinculin was quantified, and statistical significance was analyzed by comparing to the monocytes. Data are presented as the mean±SD (n=3).*, p < 0.05; **, p < 0.01; ***, p < 0.001. **(D)** Left panel, immune-score of CD68 expression in normal mucosa and gastric tumors; right panel, representative pictures of CD68 IHC staining in adjacent normal mucosa and tumor tissue of a GC patient. ***, p < 0.001. **(E)** Correlation analysis showing expression of STING and CD68 in the 200 pairs of adjacent normal mucosa and gastric cancer samples as in (A) and (D).** (F)** Immunofluorescent staining of STING (red) and CD68 (green) of a human GC sample. DAPI (blue) stained for nuclei. Scale bar represents 100 µm.

To evaluate whether STING could play a role in GC progression, we examined STING expression by IHC on a tissue array containing 200 pairs of adjacent normal and GC samples and found that STING was more highly expressed in cancer lesions than in normal tissues (Figure 1A). Further Kaplan-Meyer analysis showed that high expression of STING was associated with poorer survival of patients (Figure 1B), suggesting that STING may play a positive role in promoting GC progression. Since STING is broadly expressed in normal leukocytes, epithelial cells, and cancer cells, we performed immunoblot to examine STING expression in monocytes, macrophages, and CD3^+^ T cells derived from peripheral blood mononuclear cells (PBMCs) from healthy donors, as well as in patient-derived GC cells and HGC-27 GC cell line. We found that STING expression was more abundant in blood-cell lineages than in GC cells. Interestingly, STING was more abundantly expressed in macrophages than in CD3^+^ T cells or monocytes (Figure 1C).

Next, we examined expression of macrophage marker CD68 in the GC tissue array and found that CD68 was also highly expressed in tumor tissues compared to normal mucosa (Figure 1D), suggesting that there are more macrophages infiltrated in tumors than in normal tissues. Moreover, analyses of CD68 and STING expression in the tissue array showed a significant positive-correlation (Figure 1E), consistent with the previous conclusion that STING was most expressed in the macrophages (Figure 1C). We also examined STING and CD68 expression in pairs of normal and cancerous tissues from GC patients and found that STING was more specifically expressed in cancerous tissues compared to the broader expression of CD68 (Figure S1). We also performed immunofluorescence staining of GC samples with both anti-STING and anti-CD68 antibodies. Almost all the positive staining of STING localized in CD68^+^ macrophages (Figure 1F). Taken together, STING was highly expressed by TAMs and predicted poor survival of patients with GC.

### Both knocking-down STIING and STING activation promote macrophages polarizing into pro-inflammatory subtype

**Figure 2:**
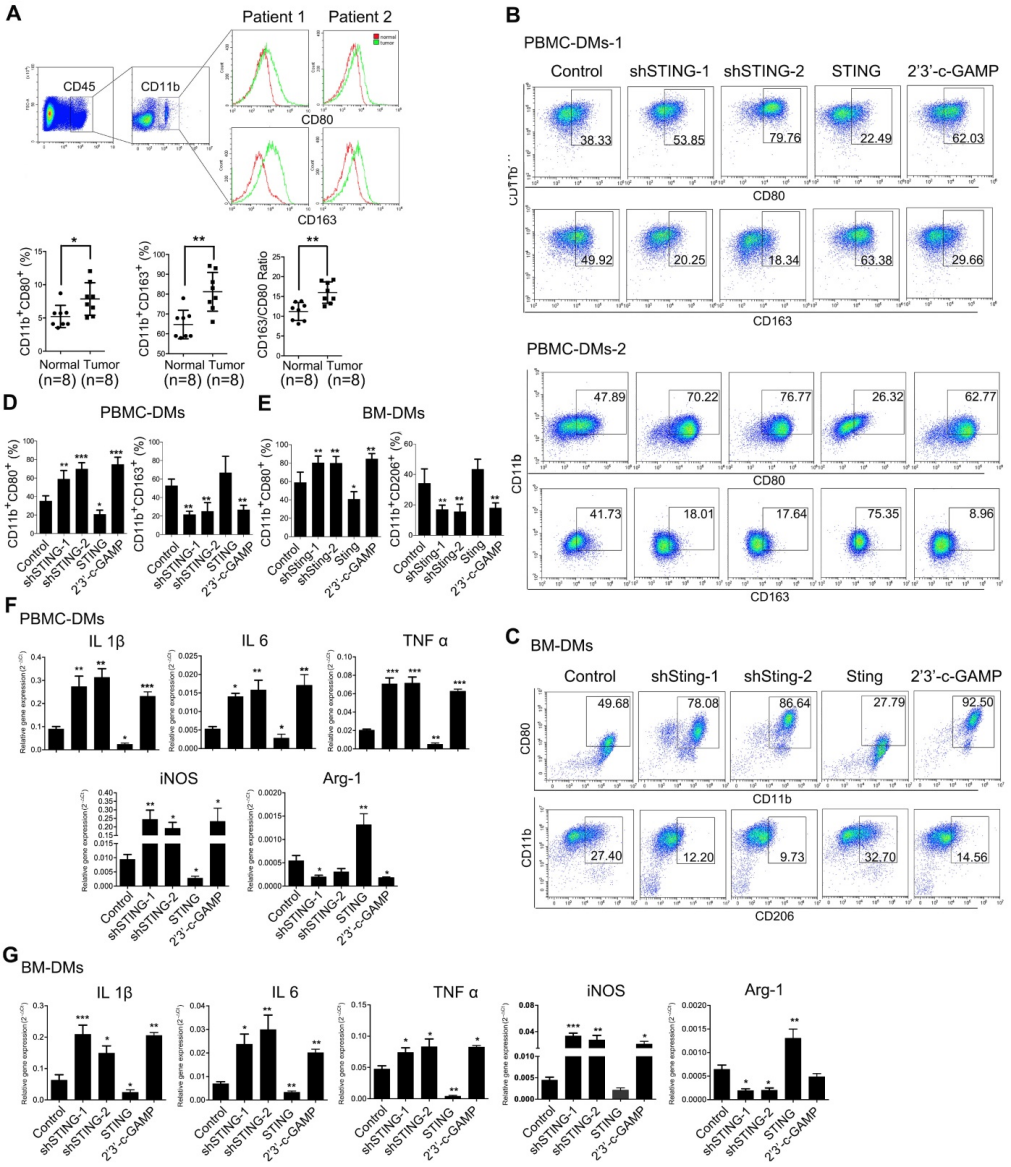
** Knocking-down STING and STING activation promote both PBMC-DMs and BM-DMs differentiating into pro-inflammatory subtype. (A)** Upper panel, flow cytometric analysis of TAMs for pro-inflammatory (CD45^+^CD11b^+^CD80^+^) and anti-inflammatory (CD45^+^CD11b^+^CD163^+^) subtypes; Lower panel, quantification of pro-inflammatory, anti-inflammatory macrophages and the corresponding ratios (n=8); *, p < 0.05; **, p < 0.01. **(B, C)** Representative flow cytometric analysis of pro-inflammatory (CD11b^+^/CD80^+^ in human (B) and mouse(C)) and anti-inflammatory macrophages (CD11b^+^/CD163^+^ in human (B) and CD11b^+^/CD206^+^ in mouse(C)) in human PBMC-DMs from two healthy donors (B) and mouse BM-DMs (C) treated as indicated. **(D, E)** Quantifications of pro-inflammatory and anti-inflammatory macrophages in human PBMC-DMs (D) and mouse BM-DMs (E) as in (B) and (C), respectively. ***, p < 0.001; **, p < 0.01; *, p < 0.05. **(F, G)** RT-PCR analysis of the macrophage subtype markers in human PBMC-DMs and mouse BM-DMs treated as indicated; Data in D, E, F and G are presented as the mean±SD (n=3).***, p < 0.001; **, p < 0.01; *, p < 0.05. Control stands for the average of undistinguishable controls of scrambled sequence (SC; for shSTING), empty vector (EV; for STING overexpression), and PBS treatment (for 2'3'-c-GAMP treatment).

To monitor immune status in the tumor microenvironment, we performed RT-PCRs to examine chemokines CXCL9, CXCL10, and IFNγ, which are important for recruiting T_H_1 cells, CD8^+^ T cells and NK cells, as well as CCL2, which attracts antigen-presenting cells (APCs), expands and activates the infiltrated effector immune cells 38. The results showed that these chemokines were lowly expressed in the tumor tissues (Figure S2A), confirming that immune status in the tumor microenvironment of GC was inhibited. Next, we analyzed 8 pairs of adjacent normal mucosa and GC samples for the macrophage subtypes. As expected, patient tumors generally contained more pro-inflammatory and anti-inflammatory macrophages than paired normal tissues, however, the ratio of anti- to pro-inflammatory macrophages was significantly increased in cancer tissues compared to adjacent normal mucosa (Figure 2A), indicating that TAMs may suppress immune responses to promote cancer progression.

We next studied the effects of STING alteration on macrophages by generating various groups of human PBMC-derived macrophages (PBMC-DMs) and mouse bone marrow-derived macrophages (BM-DMs) with either knocking-down STING or overexpressing STING by adenovirus transduction; we also activated STING in macrophages by 2'3'-c-GAMP treatment 39. Expectedly, STING expression was reduced or increased in the corresponding knocking-down or overexpression stable cell lines, while its expression was not changed by 2'3'-c-GAMP activation (Figure S2B, C). Expression of interferon-β (IFN-β) was an indication of STING activation 10. However, only STING activation by 2'3'-c-GAMP strongly enhanced IFN-β secretion, while knocking-down or overexpressing STING had little effects on IFN-β in both human and mouse macrophages (Figure S2D), indicating that STING activity and STING amount were two independent factors affecting macrophages.

Since pro-inflammatory and anti-inflammatory macrophages play contradictory anti- and pro-cancer roles, respectively, we examined the phenotypes in human and mouse macrophages with altered STING by flow cytometry. Our results showed that knocking-down STING or STING activation by 2'3'-c-GAMP both led to more abundant pro-inflammatory subtype of macrophages (CD11b^+^CD80^+^), while overexpressing STING reduced pro-inflammatory subtype in both human and mouse macrophages. Consistently, STING-overexpressing cells showed slightly more anti-inflammatory phenotype (CD11b^+^CD163^+^ in human and CD11b^+^CD206^+^ in mouse), while knocking-down STING and STING activation resulted in significantly less anti-inflammatory macrophages (Figure 2D, E, representative flow cytometry plots are shown in Figure 2B, C, Figure S2G). Moreover, we generated Sting-knockout BM-DMs based on the CRISPR-Cas9 system and received similar results (Figure S2E, F). We further performed RT-PCR analyses to examine a series of genes that could indicate the macrophage subtypes, including IL1β, IL-6, TNFα, and iNOS for pro-inflammatory subtype, and Arg-1 for anti-inflammatory subtype. The results were consistent with the flow cytometry analyses, showing that knocking-down or activating STING led to more pro-inflammatory macrophages, while STING overexpression resulted in more anti-inflammatory subtype of macrophages in both human and mouse (Figure 2F,G).

**Figure 3:**
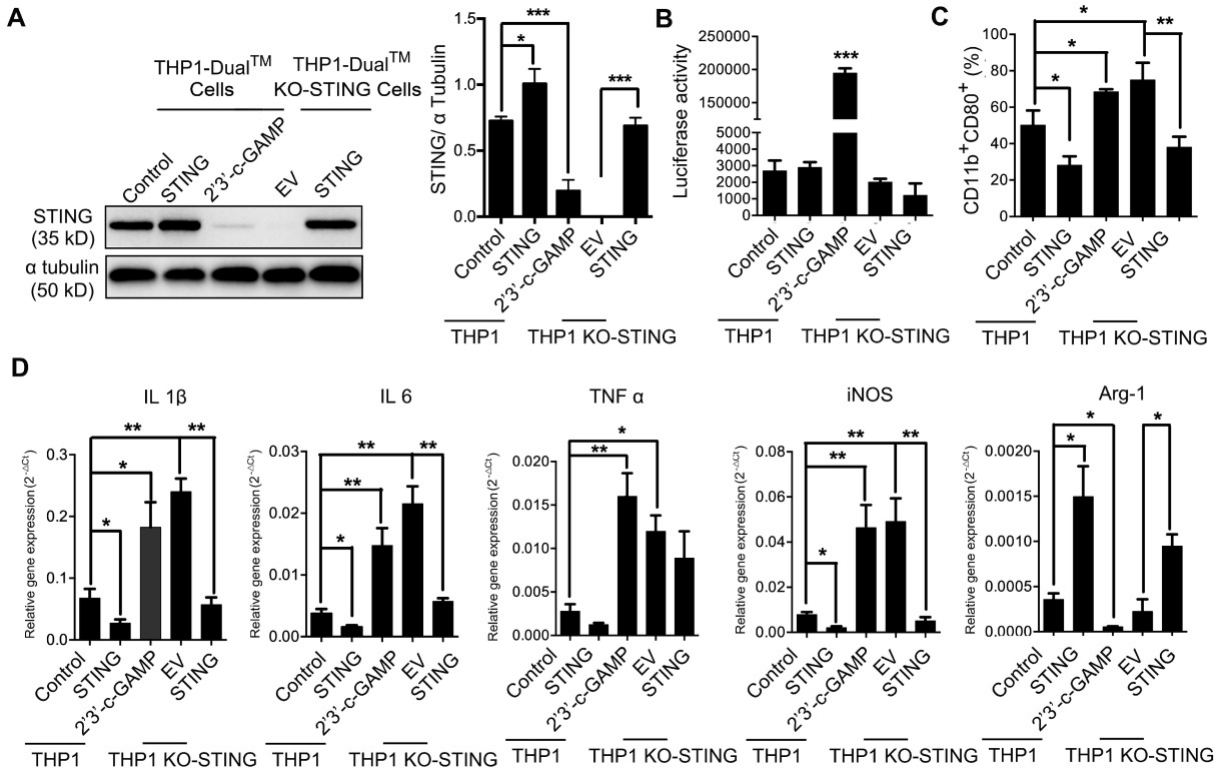
** STING knock-out and activation in THP1-derived macrophages promote pro-inflammatory subtype differentiation. (A)** Left panel, immunoblot analysis of STING expression in human THP1-derived macrophages treated as indicated. α-Tubulin was used as a loading control; right panel, the ratio of STING/α-Tubulin was quantified; ***, p < 0.001; *, p < 0.05. **(B)** Luciferase activity driven by the IFN-β promoter in THP1-derived macrophages treated as indicated. ***, p < 0.001. **(C)** Flow cytometric analysis of surface marker expression for pro-inflammatory macrophages (CD11b^+^CD80^+^) in human THP1-derived macrophages treated as indicated. *, p < 0.05.** (D)** RT-PCR analysis of the macrophage-subtype markers in stable THP1-derived macrophages treated as indicated. ***, p < 0.01; p < 0.01; *, p < 0.05. Data are presented as the mean±SD (n=3). Control stands for the average of undistinguishable controls of scrambled sequence (SC; for shSTING), and PBS treatment (for 2'3'-c-GAMP treatment), empty vector (EV) was used as a control of STING overexpression.

We also took advantage of a macrophage cell line THP1-Dual cells and its derived cell line THP1-Dual KO-STING cells with stably STING knock-out, both expressing inducible secreted Lucia luciferase reporter gene driven by IFN-β promoter 40. We overexpressed STING in both THP1- and KO-STING THP1-derived macrophages, and STING expression was restored in KO-STING cells as expected (Figure 3A). Overexpressing STING in either THP1 cells or THP1 KO-STING cells led to no difference in the luciferase activity, indicating that the transcription of STING downstream target IFN-β was not altered by STING overexpression, consistent with previous ELISA results (Figure S2C), while treatment with 2'3'-c-GAMP led to dramatic increase in luciferase activity (Figure 3B), also consistent with previous reports 10. Considering that THP1-derived macrophages barely stain of CD163 41, 42 (Figure S3), we only analyzed the staining frequency of CD11b and CD80 under different conditions. Overexpressing STING in both THP1 and THP1 KO-STING cells reduced percentage of pro-inflammatory subtype, while STING activation resulted in more abundant pro-inflammatory phenotype (Figure 3C). Interestingly, knocking-out STING in THP1 cells clearly also expanded pro-inflammatory fractions (Figure 3C, THP1-DMs KO STING vs. THP1-DMs), suggesting STING indeed inhibit differentiation of pro-inflammatory THP1 cells. A series of RT-PCR results confirmed the flow cytometry analyses (Figure 3D), also consistent with previous results (Figure 2F). Taken together, the amount of STING poses a negative effect on its activity, and both STING activation and knocking-down STING promote macrophages differentiating into pro-inflammatory subtype, while STING overexpression decreases the fraction of pro-inflammatory macrophages in human and mouse.

### Macrophages with either knocking-down STING or STING activation induce apoptosis of gastric cancer cells through JAK-IL24 pathway

**Figure 4:**
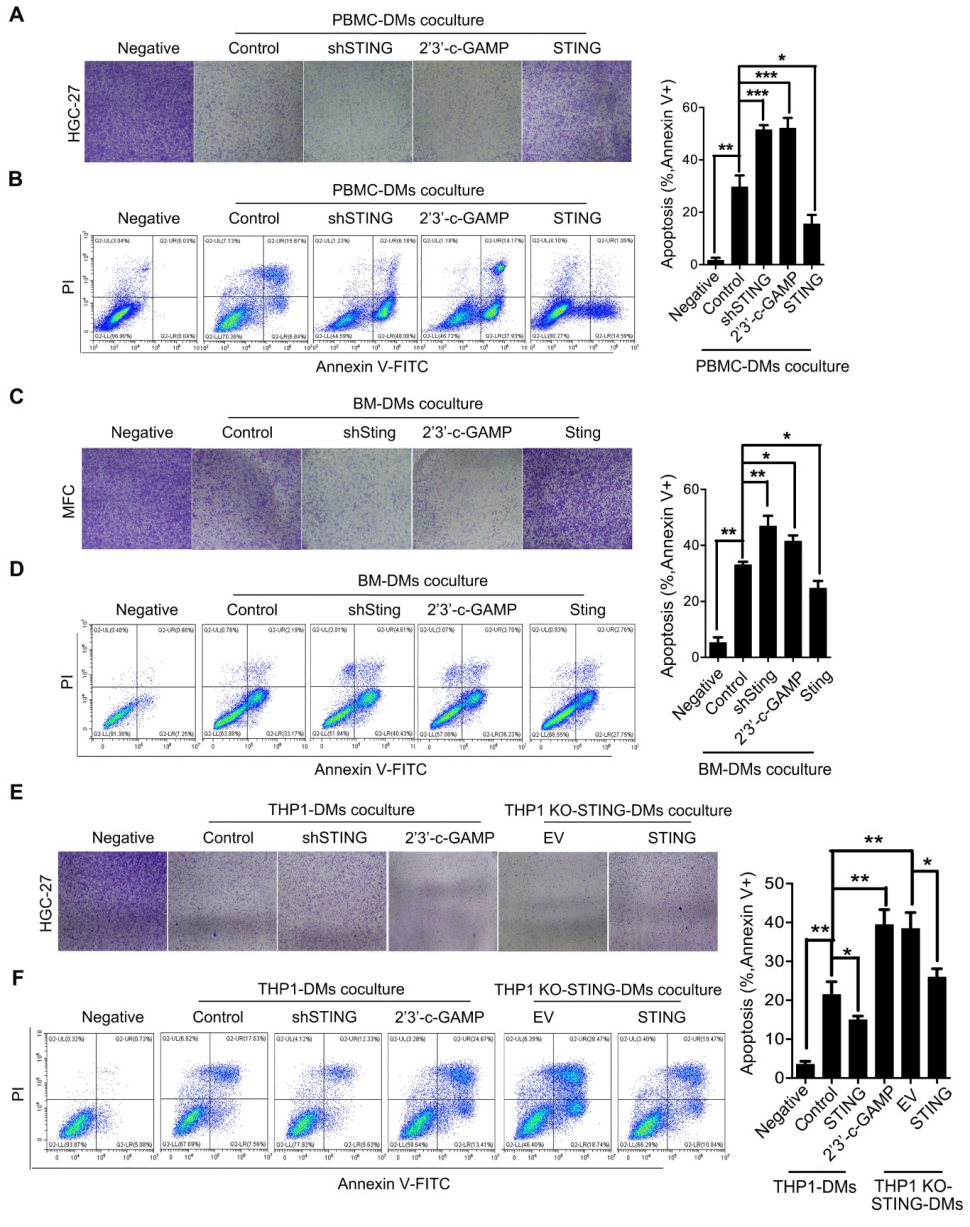
** Macrophages with knocking-down STING or STING activation have apoptoic effects on gastric cancer cells. (A)** Colony formation assay of human HGC-27 GC cells cocultured with human PBMC-DMs treated as indicated.** (B)** Left panel, representative flow cytometric plots of apoptosis markers (Annexin V^+^) in HGC-27 cells cocultured with human PBMC-DMs treated as indicated; right panel, quantification of percentage of Annexin V^+^ apoptotic cells. ***, p < 0.001; **, p < 0.01; *, p < 0.05.** (C)** Colony formation assay of mouse MFC GC cells cocultured with mouse BM-DMs treated as indicated.** (D)** Left panel, representative flow cytometric plots of apoptosis markers (Annexin V^+^) in MFC cells cocultured with mouse BM-DMs treated as indicated; right panel, quantification of percentage of Annexin V^+^ apoptotic cells. **, p < 0.01; *, p < 0.05.** (A-D)** Control stands for the average of undistinguishable controls of scrambled sequence (for shSTING), empty vector (for STING overexpression), and PBS treatment (for 2'3'-c-GAMP treatment).** (E)** Colony formation assay of human HGC-27 cells cocultured with human THP1-derived macrophages treated as indicated.** (F)** Left panel, representative flow cytometric plots of apoptosis markers (Annexin V^+^) in HGC-27 cells cocultured with human THP1-derived macrophages treated as indicated; right penal, quantification of percentage of Annexin V^+^ apoptotic cells. **, p < 0.01; *, p < 0.05. Data in B, D, F are presented as the mean±SD (n=3). **(E, F)** Control stands for the average of undistinguishable controls of scrambled sequence (SC; for shSTING), and PBS treatment (for 2'3'-c-GAMP treatment), empty vector (EV) was used as a control of STING overexpression.

We then examined the effects of macrophages with STING alteration on GC cells. We established stable cell lines from PBMC-DMs by either knocking-down STING, overexpressing STING, or treated with 2'3'-c-GAMP. We cocultured the macrophages with human HGC-27 GC cells and analyzed their viability. Interestingly, coculture with PBMC-DMs had an inhibitory effect on the proliferation of HGC-27 cells, while knocking-down STING or STING activation further enhanced this inhibitory effect. However, STING overexpression totally rescued the inhibition on HGC-27 proliferation (Figure 4A). The inhibitory effects of macrophages on GC cells might be through cell cycle arrest or apoptosis induction, so we analyzed cell cycle and apoptosis progression of HGC-27 cells cocultured with different groups of PBMC-DMs. We found that cell cycle progression of HGC-27 cells in different groups were not changed (Figure S4D), while apoptosis of HGC-27 cells were significantly increased when cocultured with PBMC-DMs (Figure 4B), Furthermore, both knocking-down STING and STING activation dramatically increased apoptosis of HGC-27 cells. However, overexpression of STING reduced apoptosis of cancer cells compared to those cocultured with unaltered macrophages (Figure 4B). Macrophage-induced apoptosis was also confirmed by immunobloting of cleaved and total PARP expression (Figure S4A).Mouse GC cell line MFC also showed increased apoptosis when cocultured with mouse BM-DMs, and apoptosis was further enhanced by either knocking-down Sting or 2'3'-c-GAMP treatment in BM-DMs, yet Sting overexpression reduced the apoptosis-inducing effects of macrophages (Figure 4C, D and Figure S4B). Cell cycle analyses of MFC cells also showed no differences between those cultured alone or cocultured with different groups of BM-DMs (Figure S4D).

We also examined the apoptosis-inducing effects of THP1-derived macrophages on HGC-27 GC cells. Coculture with either THP1 cells or THP1 KO-STING cells showed significant effects in inducing apoptosis of HGC-27 cells, yet KO-STING cells had more profound killing effects (Figure 4E, F). Overexpression of STING reduced the apoptosis-inducing ability of both cell lines, while STING activation increased apoptosis induced by THP1-derived macrophages to the similar level as THP1 KO-STING cells (Figure 4E, F and Figure S4C).

Since macrophages can work as APCs to influence T cells, we also examined the effects of STING-altered macrophages on differentiation of T cells by cocultuing PBMC-DMs with altered STING expression or activity together with CD3^+^ T cells from PBMC (Figure S4E), and we found that distribution of CD4/CD8 T cells was not changed by STING alteration in macrophages (Figure S4F, G).

**Figure 5:**
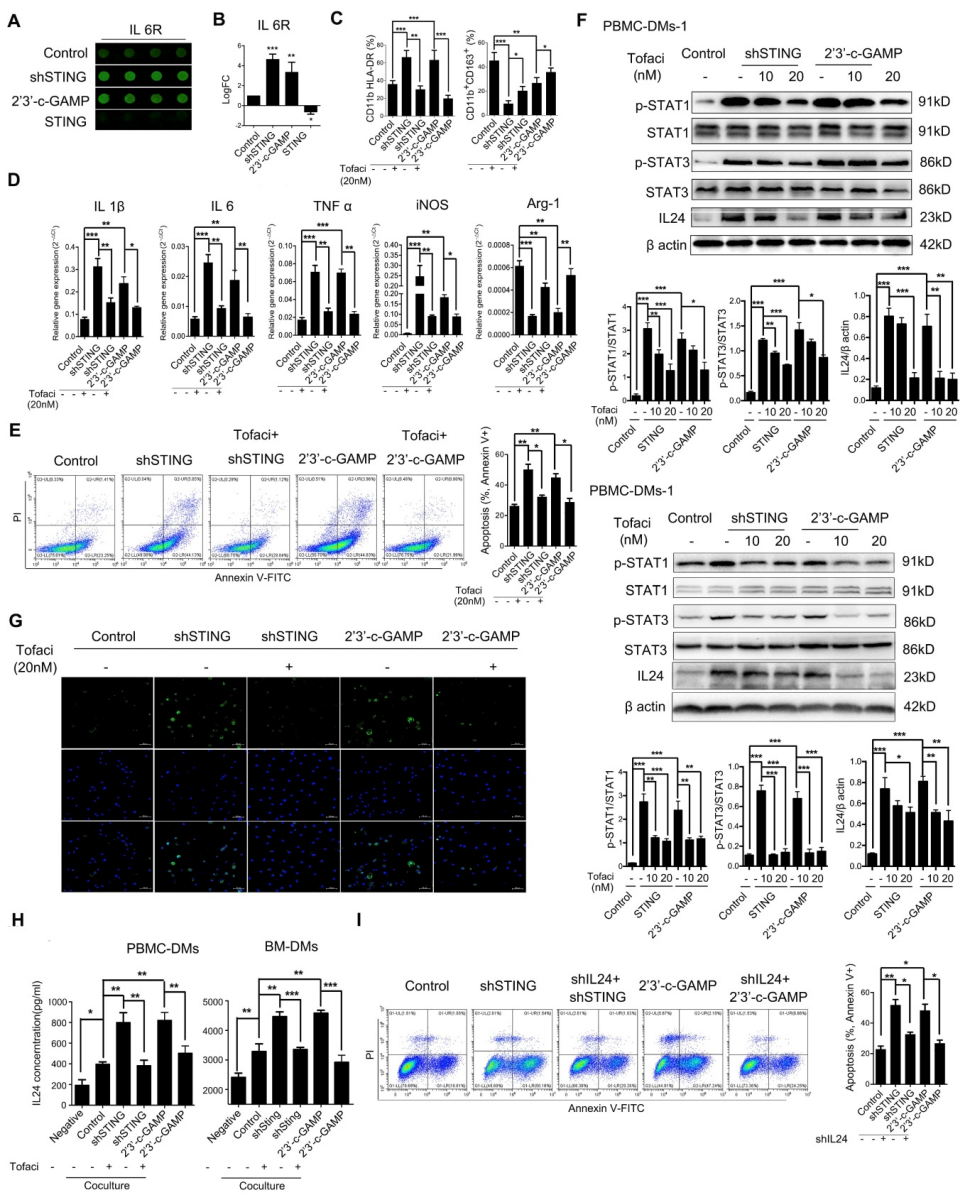
** STING knocking-down and activation regulate macrophage differentiation through JAK-IL24 pathway. (A)** Representative pictures of IL6R in a cytokine array analysis of supernatants from PBMC-DM cultures treated as indicated, and quantified in **(B)**; ***, p<0.001; **, p< 0.01.** (C)** Flow cytometric analysis of surface marker expression for pro-inflammatory macrophages (CD11b^+^/HLA-DR^+^) and anti-inflammatory macrophages (CD11b^+^/CD163^+^) in human PBMC-DMs treated as indicated. ***, p < 0.001; **, p < 0.01; *, p < 0.05.** (D)** RT-PCR analysis of the macrophage subtype-markers in human PBMC-DMs treated as indicated. ***, p < 0.001; **, p < 0.01; *, p < 0.05.** (E)** Left panel, representative flow cytometric plots of apoptosis markers (FITC-Annexin V^+^) in human PBMC-DMs treated as indicated; right panel, quantification of percentage of Annexin V^+^ apoptotic cells; **, p < 0.01; *, p < 0.05.** (F)** Immunoblot analysis of phospho-STAT1, total STAT1, phospho-STAT3, total STAT3, IL24 expression in PBMC-DMs from two healthy donors treated as indicated; β-Actin was used as a loading control; quantification of the immunoblot analysis of PBMC-DMs from each healthy donor was below the bands; ***, p < 0.001; **, p < 0.01; *, p < 0.05. **(G)** Immunofluorescent staining of IL24 (green) in human PBMC-DMs. DAPI (blue) stained for nuclei. Scale bar represents 50 µm.** (H)** IL24 expression measured by ELISA in supernatants of human PBMC-DMs (left) or mouse BM-DMs (right) cultures treated as indicated. ***, p < 0.001; **, p < 0.01; *, p < 0.05. **(I)** Left panel, representative flow cytometric plots of apoptosis markers (FITC-Annexin V^+^) in human PBMC-DMs treated as indicated; right panel, quantification of percentage of Annexin V^+^ apoptotic cells; **, p < 0.01; *, p < 0.05. Data in B,C, D, E and F are presented as the mean±SD (n=3). (A-E, H) Control stands for a representative sample of undistinguishable controls of scrambled sequence (for shSTING), empty vector (for STING overexpression), and PBS treatment (for 2'3'-c-GAMP treatment). (F, G, I) Control stands for a representative sample of undistinguishable controls of scrambled sequence (for shSTING and/or shIL24) and PBS treatment (for 2'3'-c-GAMP treatment).

To understand how PBMC-DMs induce apoptosis of cancer cells in the coculture system, we performed cytokine arrays comparing the supernatants from PBMC-DMs with altered expression or activity of STING and control PBMC-DMs. We found numerous changed cytokines, and pathway analyses using the altered cytokines showed that JAK-STAT signaling pathway was enriched in the secreted proteins from both STING knocking-down and STING activation macrophages (Figure S5A). As one of the most significantly changed cytokines, IL6R caught our attention (Figure 5A and B), since it has been reported that IL-6/IL-6R complex plays a pivotal role in the JAK-STAT pathway during immune responses and cancer progression 43. We then utilized a small-molecule JAK inhibitor Tofacitinib 44, which could significantly reduce the increased pro-inflammatory phenotypes induced by either knocking-down STING or activating STING (Figure 5C, representative flow cytometry diagrams in Figure S5B). Likewise, Tofacitinib could also rescue the anti-inflammatory phenotypes also induced by STING alteration (Figure 5C, representative flow cytometry diagrams in Figure S5B). RT-PCR examination with macrophage subtype markers also confirmed that JAK pathway inhibition could rescue the pro-inflammatory-inducing and anti-inflammatory-inhibiting effects of either knocking-down STING or STING activation (Figure 5D). Therefore, knocking-down STING or 2'3'-c-GAMP treatment might activate IL-6R-JAK-STAT pathway to promote polarization of pro-inflammatory macrophages.

To see whether IL-6R-JAK-STAT pathway plays a role in apoptosis-induction of PBMC-DMs with STING knocked-down or activation, we performed flow cytometry analyses of HGC-27 cells cocultured with different groups of PBMC-DMs treated with Tofacitinib. Our results showed that JAK inhibition could rescue the apoptosis-induction of STING-altered macrophages (Figure 5E, Figure S5C), suggesting that either STING knocked-down or activation leads to activation of JAK pathway. One of the IL-6R-JAK-STAT downstream effectors is IL24, which regulates cell apoptosis by binding to IL-20R1/IL-20R2 and IL-20R2/IL-22 receptor complexes 45. Thus, we performed immunoblot and immunofluorescence analyses to examine the endogenous activation of JAK pathway and expression of IL24 in PBMC-DMs with altered STING. Interestingly, knocking-down STING and STING activation both activated phosphorylation of STAT1 and STAT3, both of which were inhibited dose-dependently by Tofacitinib treatment (Figure 5F). Of note, IL24 was also induced by knocking-down STING and STING activation (Figure 5F), which might be the core mediator to induce apoptosis in cancer cells. Moreover, immunofluorescence staining of PBMC-DMs with anti-IL24 antibody confirmed the enhanced IL24 expression in macrophages with knocking-down STING or 2'3'-c-GAMP treatment, while the enhancement was reduced by Tofaticinib treatment (Figure 5G). Next, we also performed ELISA to examine the secretion of IL24 by macrophages with various treatments cocultured with GC cells. First, macrophages indeed increased IL24 secretion cocultured with both human and mouse cancer cells; secondly, knocking-down STING and 2'3'-c-GAMP treatment both further increased IL24 content in supernatants, yet treatment with JAK inhibitor Tofacitinib fully rescued the amount of IL24 to control level; thirdly, overexpressing STING inhibited IL24 secretion by macrophages. We further confirmed the essential role played by IL24 by establishing stable shIL24-expressing macrophages, and showed that knocking-down IL24 could rescue the apoptosis-inducing effects of macrophages with either knocking-down STING or 2'3'-c-GAMP treatment in human and mouse (Figure 5I, Figure S5C, D).Taken together, macrophages with altered STING polarize into pro-inflammatory phenotype and induce apoptosis of GC cells through activation of IL-6R-JAK-STAT pathway and induction of its downstream target IL24.

### Macrophages with either knocking-down STING or STING activation have therapeutic effects on *in vivo* gastric cancer models

**Figure 6:**
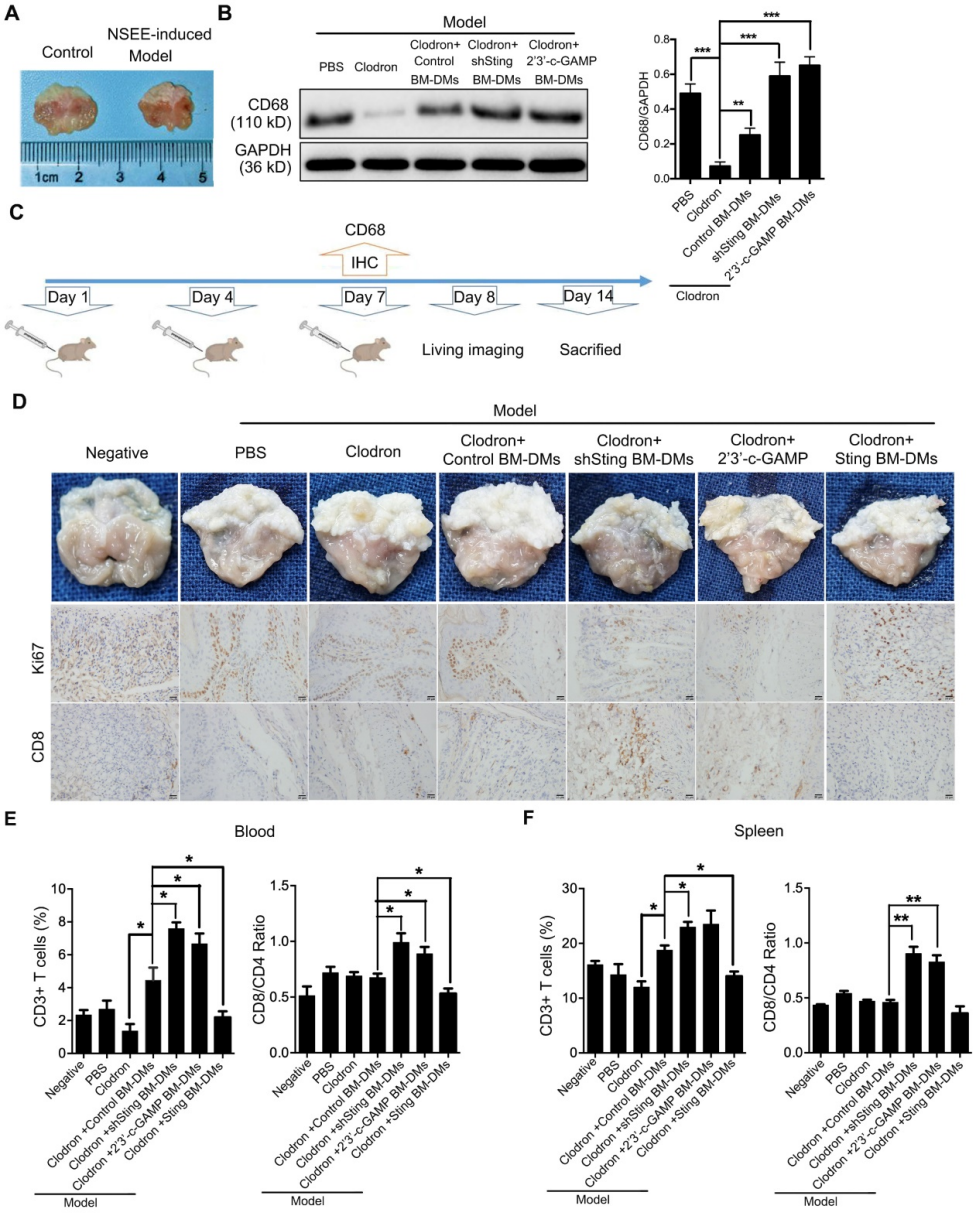
** Macrophages with Sting knocking-down or activation have killing effects on cancer cells of spontaneous gastric tumors in mice. (A)** Visual examination and tumor development in stomachs of mice treated as indicated.** (B)** Scheme showing the time course of treatments in mice.** (C)** Left panel, immunoblot analysis of CD68 expression in gastric tissues from mice treated as indicated; GAPDH was used as a loading control; right panel, the ratio of CD68/GAPDH was quantified, and statistical significance was analyzed by comparing to the Clodron-treated samples; **, p < 0.01; ***, p < 0.001. Control stands for a representative sample of undistinguishable controls of scrambled sequence (for shSTING) and PBS treatment (for 2'3'-c-GAMP treatment). **(D)** Upper panel, visual examination and tumor development in stomachs of mice treated as indicated; lower panel, representative pictures of Ki67 and CD8 IHC staining in gastric normal and tumor tissues corresponding to the upper panel. Scale bars represent 20 µm.** (E)** Quantification of surface marker expression for total T cells (CD3^+^) and ratio of effector/helper T cells (CD8^+^/CD4^+^) in the blood of mice treated as indicated. *, p < 0.05.** (F)** Quantification of surface marker expression for total T cells (CD3^+^) and ratio of effector/helper T cells (CD8^+^/CD4^+^) in the spleens of mice treated as indicated. **, p < 0.01. *, p < 0.05. Data in B, E and F are presented as the mean±SD (n=10). **(D-F)** Control stands for a representative sample of undistinguishable controls of scrambled sequence (for shSTING), empty vector (for STING overexpression), and PBS treatment (for 2'3'-c-GAMP treatment).

The *in vitro* experiments showed that macrophages with knocking-down STING or STING activation could induce apoptosis of GC cells, so we next tested the therapeutic potential of macrophages in mouse models. First, we induced spontaneous GC in mice by feeding N-nitroso-sarcosine-ethyl (NSEE) for 16 weeks, and stomachs of NSEE-induced mice exhibited numerous cancerous nodules (Figure 6A). We then depleted endogenous macrophages of mice by injecting clodronated liposomes twice in a week intraperitoneally; PBS was delivered as a negative control (Figure 6B). To confirm the effects of liposome-induced macrophage depletion, we performed IHC of the macrophage marker CD68, which was completely lost in liposome-injected mice (Figure S6A). 7 days later, BM-DMs, which engulfed pHrodo^TM^ red bioparticles, were injected intraperitoneally (Figure 6B). 1 day after, we could clearly observe red fluorescence in mouse stomachs, indicating the successful transplantation and significant infiltration of macrophages into tumor tissues (Figure S6B). 6 days later, stomachs were examined (Figure 6B).

We performed immunoblot analyses of mouse bone marrow cells to show that macrophage marker CD68 was almost lost after clodronated liposome injection, while transplantation of BM-DMs rescued CD68 expression regardless STING condition (Figure 6C). Visual examination of stomachs after treatments showed that NSEE indeed induced cancer development in the stomachs by increasing cell proliferation, shown by upregulation of Ki67 (Figure 6D). Mice treated with clodronated liposomes depleted macrophages, promoting cancerous nodule growth (Figure 6D). Transfusion with exogenous macrophages had little effects on cancer development; however, transfusion of Sting-knocking-down or Sting-activated macrophages significantly reduced number of nodules, also increased tumor-infiltrating CD8^+^ T cells. In contrast, Sting-overexpressing macrophages had little effects on cancer progression and CD8^+^ T cell recruitment (Figure 6D). The deterioration of mice could also been seen by the relatively fast weight-loss of mice injected with PBS or Sting-overexpressing macrophages (Figure S6C). We also examined T cell activation in the blood and spleens of mice. NSEE-induced cancer did not simultaneously increase numbers of CD3^+^ T cells in the circulation, yet macrophages depletion reduced total T cells in blood (Figure 6E). Transfusion with exogenous macrophages rescued numbers of T cells, which was further enhanced by macrophages with knocking-down Sting or activated Sting (Figure 6E). However, Sting-overexpressing macrophages lost the rescue effects on the frequency of T cells ((Figure 6E, representative flow cytometry diagrams in Figure S6D). To reveal the activity of T cells, we performed a series of flow cytometry analyses comparing the ratio of CD8^+^/CD4^+^ T cells, which indicates the relative effectiveness of T cells. Our results showed that only transfusion of macrophages with Sting knocking-down or Sting activation could enhance the ratio of CD8^+^/CD4^+^, while macrophages with overexpressing Sting had no effects on T cells (Figure 6E, representative flow cytometry diagrams in Figure S6D). We also analyzed T cell abundance and CD8^+^/CD4^+^ distribution in the spleens of mice with aforementioned treatments, and received similar results (Figure 6F, representative flow cytometry diagrams in Figure S6D and E), suggesting that macrophages with knocking-down Sting or Sting activation have profound anti-tumor effects, along with enhancing the abundance and effectiveness of T cells in spontaneous GC mouse models.

**Figure 7:**
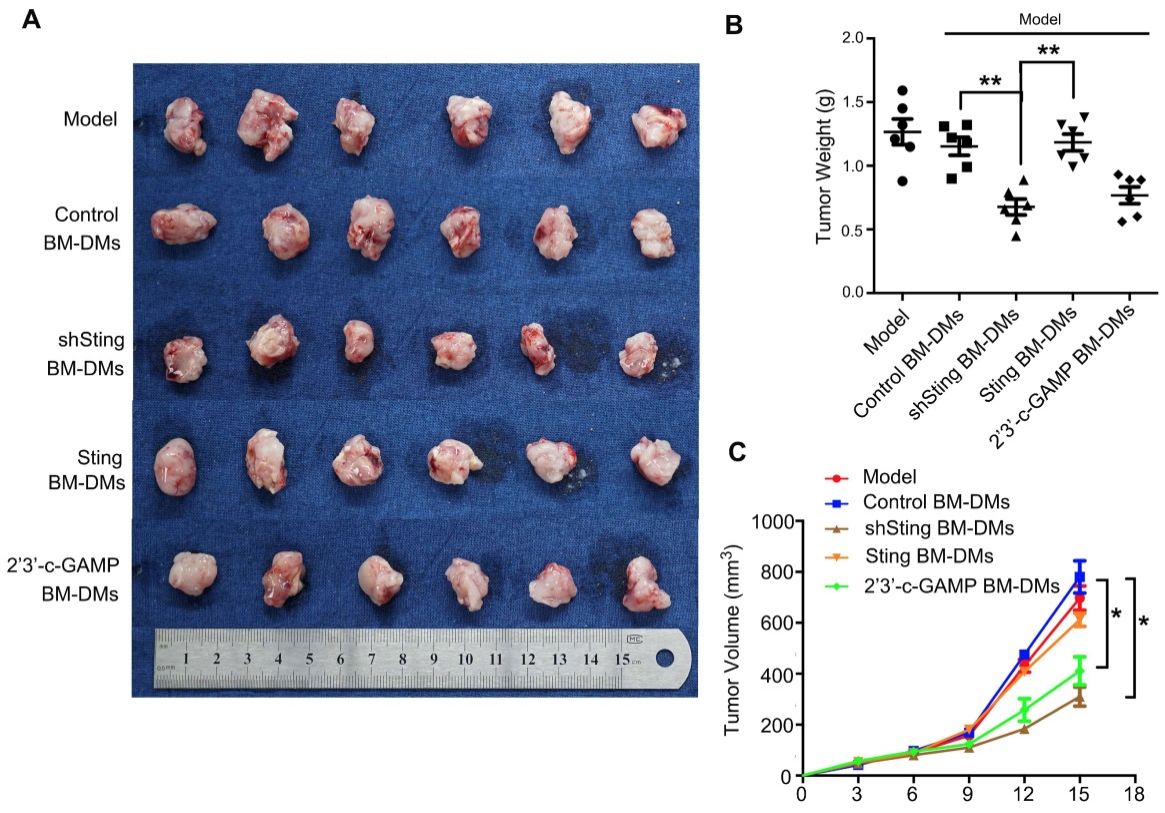
** Macrophages with Sting knocking-down or activation have killing effects on cancer cells of xenografted gastric tumors in mice. (A)** Visual examination of isolated tumors from mice injected subcutaneously with MFC cells and treated as indicated. **(B)** Tumor weight in mice after indicated treatments; **, p < 0.01. **(C)** Tumor volume in mice with indicated treatments; *, p < 0.05. Data in B,C are presented as the mean±SD (n=6). Control stands for a representative sample of undistinguishable controls of scrambled sequence (for shSting), empty vector (for Sting overexpression), and PBS treatment (for 2'3'-c-GAMP treatment).

We also examined therapeutic effects of Sting-altered macrophages in xenograft mouse models. We first transplanted mouse MFC GC cells subcutaneously, and treated them with macrophages that were altered Sting expression or activity. Our results confirmed the anti-cancer effects of macrophages with knocking-down Sting or activated Sting in the previous spontaneous GC model; mice treated with these macrophages showed dramatic reduced burden in tumor weights and volumes (Figure 7). Taken together, macrophages with knocking-down Sting or Sting activation have profound anti-tumor effects in GC mouse models.

## Discussion

Tumor cells are notoriously non-immunogenic and acquire properties that enable them to evade the immunosurveillance system. Analyses from adjacent normal and GC samples confirmed that immune-related status was passive in the tumor microenvironment compared to normal tissues, as reflected by scarce expression of chemokines CXCL9, CXCL10, and CCL2, leading to IFN-γ restriction. Hence, reversing the suppressive tumor microenvironment is an important strategy for immunotherapy. The innate immune system plays a key role in initiating adaptive immunity, and thus influences tumor development and immunotherapy. TAMs are a crucial component of the innate immune system, and the ratio of pro-inflammatory to anti-inflammatory TAMs in GC is closely related to survival of patients 4. Taken together, we hypothesized that immunotherapy targeting TAMs may be effective in GC patients.

STING is a core player in the innate immune system 3, and we first identified macrophages as the major source of STING compared with other cell types, including cancer cells, monocytes, and T cells. Besides, a significant positive-correlation existed between macrophage marker CD68 and STING in a tissue array of 200 pairs of patient samples. Moreover, expression of STING was associated with poor survival of these GC patients. STING may be an inhibitory component in the cancer immune microenvironment, since STING activation could induce immune tolerogenic state by inducing inhibitory signal IDO to protect cancer cells from immune attack 46, 47. The expression of STING is also positively correlated with the abundance of regulatory T cells (Tregs) in patients with tongue squamous carcinoma 48. Tumor cell-derived microparticles could also activate the STING pathway to polarize TAMs to anti-inflammatory subtype, thus to promote tumor progression 49. Models including STING or cGAS could more precisely predict radiographic and pathological therapy responses, respectively 50. It has also been reported that STING activation in T cells also triggers canonical inflammatory IFN production, but prevents T cell proliferation and simulates T cell death events 51-53. In contrast to previous studies, our data deepened the functional investigation of STING by distinguishing the amount from the activity of STING, by showing that both knocking-down STING and STING activation promoted macrophages polarizing into pro-inflammatory subtype, while overexpressing STING reduced the percentage of pro-inflammatory macrophages in both human and mouse.

Although it is contradictory concerning the similar effects of knocking-down STING and STING activation, we noticed that activated STING was transferred from endoplasmic reticulum to the periphery of the nucleus, leading to the reduced level of STING in endoplasmic reticulum, which was also found in cells with knocking-down STING (data not shown). Therefore, we hypothesized that STING may interact with certain proteins in the endoplasmic reticulum, which is released from binding after STING knocking-down or activation, leading to pro-inflammatory polarization of macrophages. It is known that pro-inflammatory-subtype macrophages generally play an anti-cancer role during cancer progression 9. In the coculture system, knocking-down STING or STING activation in macrophages indeed had an inhibitory effect on human and mouse GC cells, resulting in apoptosis induction. The anti-tumor effects of STING-manipulated macrophages were further confirmed in the *in vivo* studies. Consistent with *in vitro* experiments, macrophages with knocking-down Sting or Sting activation decreased cancerous nodules and expression of Ki67, as well as enhanced the abundance and priming of T cells in spontaneous GC mouse models. The MFC-derived xenograft mouse models also confirmed the anti-cancer effects of transplanting macrophages with Sting manipulation. Taken together, both spontaneous and xenograft mouse models provide valuable platform for studying the effects of targeting macrophages during GC treatment.

The tumor tissues of GC patients contained more macrophages (both pro-inflammatory and anti-inflammatory) than paired normal tissues, while the ratio of pro-inflammatory to anti-inflammatory was significantly decreased in cancer tissues. STING knocking-down and 2'3'-c-GAMP treatment both enhanced the fraction of pro-inflammatory macrophages and decreased anti-inflammatory macrophages; in contrast, overexpressing STING reduced the pro-inflammatory macrophages. Therefore, STING knocking-down or activation in macrophages may exhibit optimistic therapeutic effects in GC patients. Macrophages have been reported to present antigens to T cells, thus to activate T cells to CD8^+^ T cells 53; however, coculture of macrophages with STING alteration has little effects on T cell differentiation, which might be due to the lack of presentable antigens in the coculture system. In contrast, transfusion of macrophages could successfully activate and recruit CD8^+^ T cells in the spontaneous GC mouse model. Therefore, our data established a role for activating STING in the control of GC progression through not only promoting pro-inflammatory macrophage polarization, but also recruiting effective T cells.

Moreover, different from STING activation, knocking-down STING induced apoptosis of GC cells without increasing IFN-β. Repolarization of TAMs from anti- to pro-inflammatory phenotype is associates with increased TNF-α, but attenuated TGF-β 23. JAK/STAT signaling pathway is also closely associated with macrophage polarization 54, 55. IL-6 secreted by TAMs is highly correlated with the occurrence and development of hepatic carcinoma via activating STAT3 pathway 56. In our study, JAK-STAT signaling pathway was enriched in the secreted proteins from STING-altered macrophages, indicating that JAK-STAT signaling pathway may play an important role in the pro-inflammatory polarization and apoptosis-induction of macrophages. Since IL-6/IL-6R complex is a pivotal upstream mediator of the JAK-STAT pathway during immune responses and cancer progression 43, and the cytokine analyses also showed that IL6R was indeed one of the most significantly changed cytokines, we hypothesized that JAK/STAT pathway is downstream of knocking-down STING and STING activation through IL6R. We took advantage of pharmacological JAK inhibitor Tofacitinib to study the function of JAK-STAT signaling pathway in STING-altered macrophages, and our results showed that Tofacitinib significantly reduced the increased pro-inflammatory phenotypes that were induced by either knocking-down STING or activating STING. We further set out to investigate the essential downstream factor of IL-6R-JAK-STAT signaling pathway, which could induce GC cell apoptosis. As a potential candidate, IL24 is mainly produced by epithelial and innate immune cells and has the ability to promote apoptosis by binding to IL-20R1/IL-20R2 and IL-20R2/IL-22 receptor complexes 57, 58. Interestingly, knocking-down STING and STING activation both increased IL24 concentration in supernatants of altered macrophages, which could be rescued by treatments with Tofacitinib, while knocking-down IL24 could also rescue the apoptosis-inducing effects of macrophages, confirming that IL24 is the essential downstream mediator of STING pathway activation. Therefore, our data showed that controlling STING-dependent cytokine production, especially IL24, could have beneficial effects on GC treatment.

Taken together, the present investigation included both *in vitro* and *in vivo* experiments to elucidate the roles played by STING in macrophages. Our data examined how macrophages contributed to destroy cancer cells and provided a therapeutic strategy to stimulate anti-tumor immunity. Macrophages with altered STING induced pro-inflammatory phenotype and apoptosis of GC cells through activation of IL-6R-JAK-STAT pathway and its downstream target IL24. Therefore, manipulating STING pathway to modulate TAMs might be a promising strategy for GC immunotherapy. Further study of STING-binding proteins is also necessary to explore the mechanisms of downstream signaling pathways and critical cytokines.

## Supplementary Material

Supplementary figures and tables.

## Abbreviations

STING: synthase-stimulator of interferon genes
ISGs: IFN-stimulated genes
TAMs: tumor-associated macrophages
CAR-T: chimeric antigen receptor T cell
ICIs: immune checkpoint inhibitors
PRRs: pattern recognition receptors
NK: natural killer
APCs: antigen-presenting cells
GEPIA: Gene Expression Profiling Interactive Analysis
BM-DMs: bone marrow-derived macrophages
PBMC-DMs: peripheral blood mononuclear cells-derived macrophages
iNOS: inducible nitric oxide synthase
TNF: tumor necrosis factor
GC: Gastric cancer
NSEE: N-nitroso-sarcosine-ethyl
IDO: indoleamine 2,3-dioxygenase
