# Targeting the STING pathway in tumor-associated macrophages regulates innate immune sensing of gastric cancer cells: Erratum

**DOI:** 10.7150/thno.114260

**Published:** 2025-04-19

**Authors:** Lei Miao, Jingjing Qi, Qi Zhao, Qi-Nian Wu, Da-Liang Wei, Xiao-Li Wei, Jia Liu, Jun Chen, Zhao-Lei Zeng, Huai-Qiang Ju, Hui-yan Luo, Rui-Hua Xu

**Affiliations:** 1 State Key Laboratory of Oncology in South China, Sun Yat-sen University Cancer Center, Collaborative Innovation Center for Cancer Medicine, Guangzhou 510060, China.; 2 Department of Pediatric Surgery, Guangzhou Women and Children's Medical Center, Guangzhou Medical University, Guangzhou, Guangdong, China.; 3 Department of Pathology, Sun Yat-sen University Cancer Center, State Key Laboratory of Oncology in South China, Collaborative Innovation Center for Cancer Medicine, Guangzhou 510060, China.; 4 Department of Medical Oncology, Sun Yat-sen University Cancer Center, State Key Laboratory of Oncology in South China, Collaborative Innovation Center for Cancer Medicine, Guangzhou 510060, China.; 5 Zhongshan School of Medicine, Sun Yat-sen University, Guangzhou 510060, China.

The authors regret that incorrect pictures were accidentally displayed during data preparation, including colony formation photo, flow cytometry scatter diagram and immunoblots in Figure 2B, Figure 4, and Supplementary figure. The authors confirm that these corrections do not change the statistical analyses or conclusions of the article. The authors apologize for any inconvenience that the errors may have caused.

## Figures and Tables

**Figure A:**
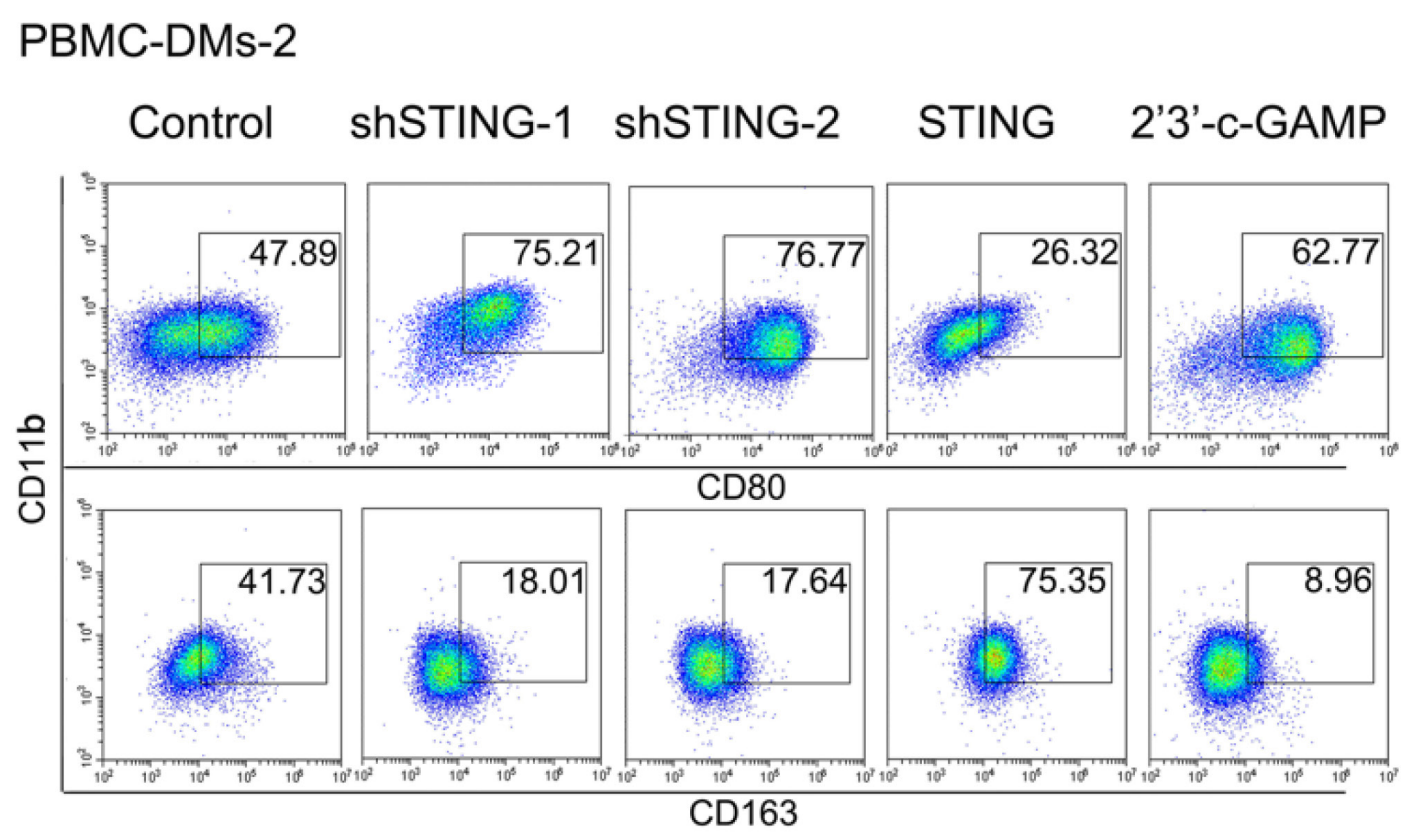
** Corrected Figure 2B.** Representative flow cytometric analysis of pro-inflammatory (CD11b^+^/CD80^+^) and anti-inflammatory macrophages (CD11b^+^/CD163^+^) in human PBMC-DMs from two healthy donors treated as indicated.

**Figure B:**
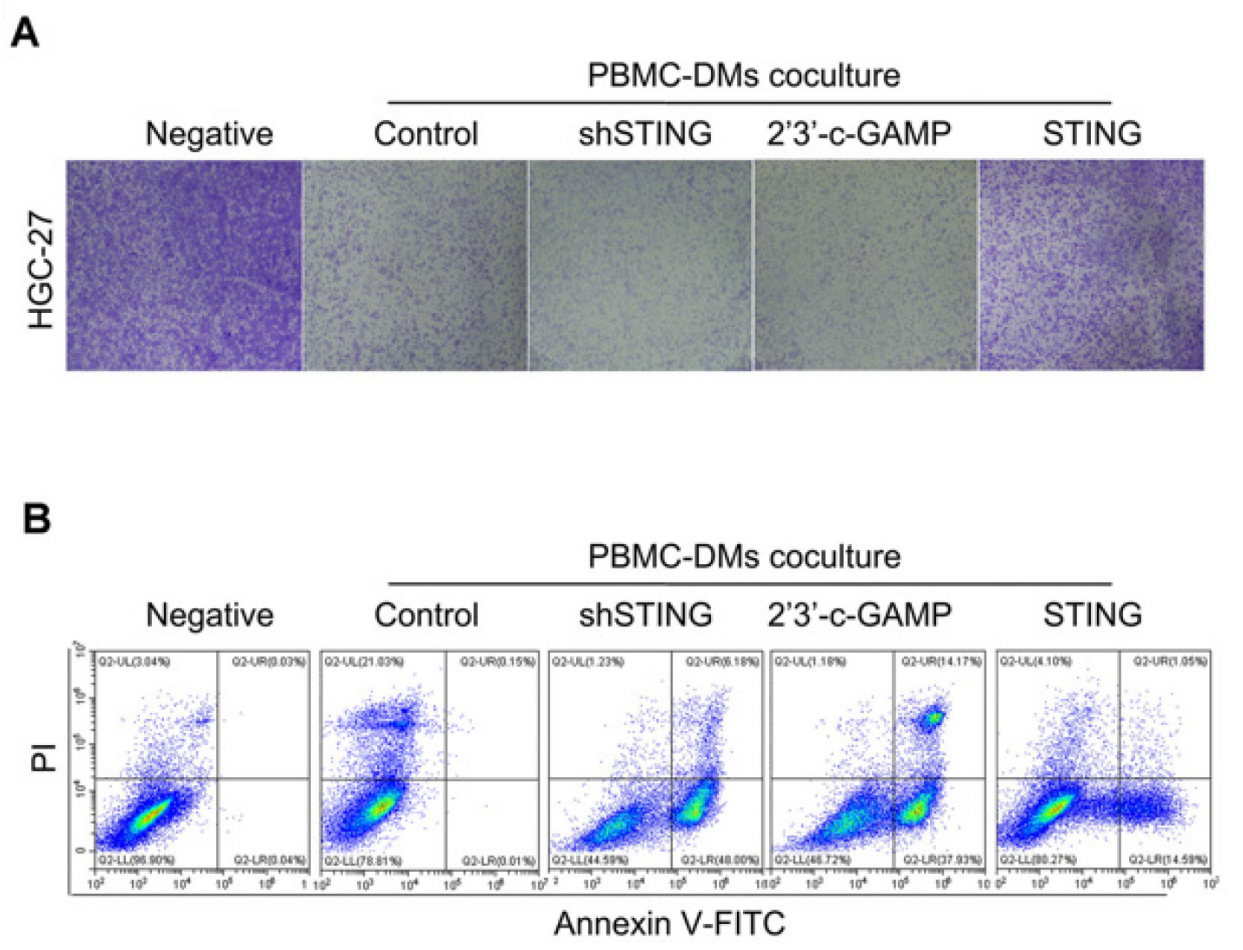
** Corrected Figure 4A.** Colony formation assay of human HGC-27 GC cells cocultured with human PBMC-DMs treated as indicated. **(B)** Left panel, representative flow cytometric plots of apoptosis markers (Annexin V+) in HGC-27 cells cocultured with human PBMC-DMs treated as indicated.

**Figure C:**
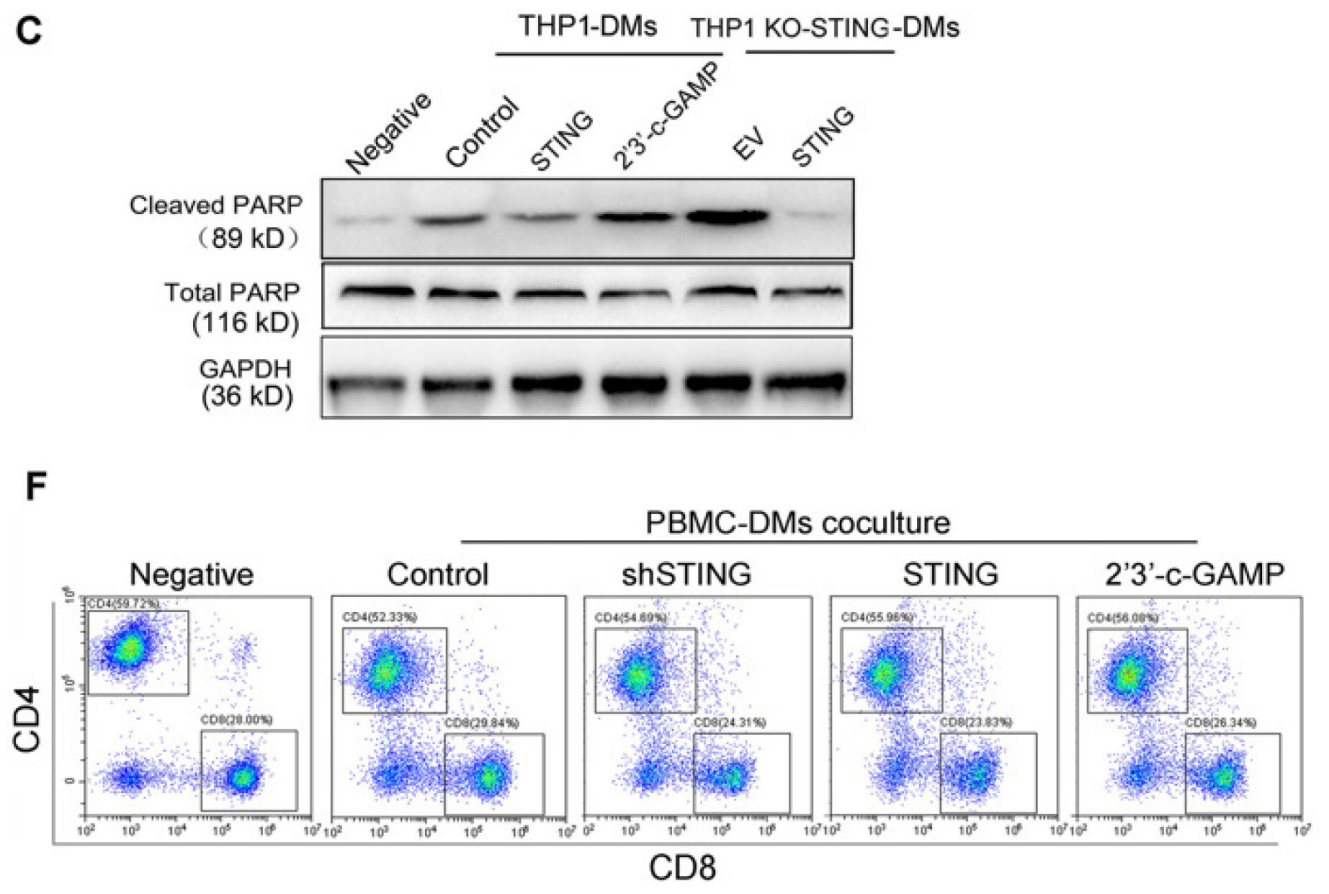
** Corrected Figure S4. (C)** Immunoblot analysis of cleaved-PARP and total PARP expression in human THP1-DMs treated as indicated. GAPDH was used as a loading control. **(F)** Representative flow cytometric plots of CD4/CD8 T cells in cocultures of human T cells with human PBMC-DMs treated as indicated.

**Figure D:**
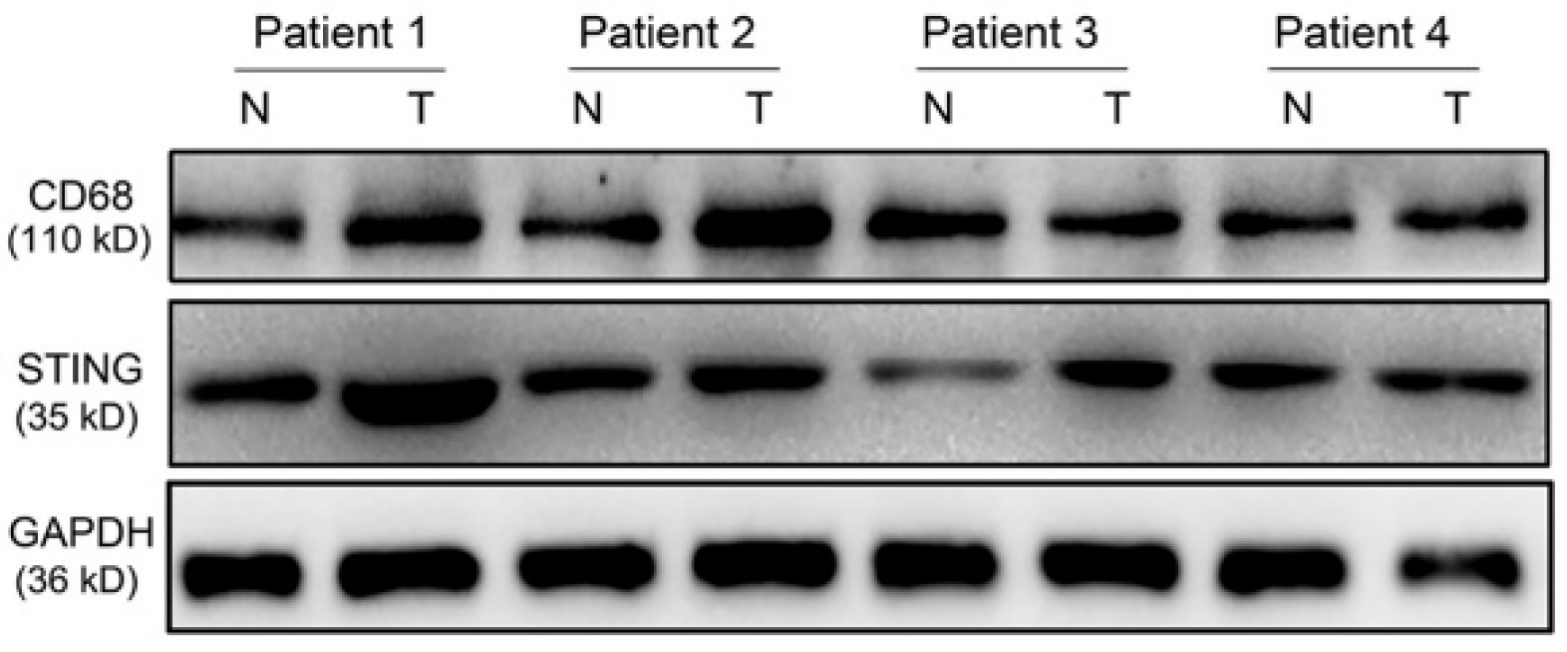
** Corrected Figure S1.** Immunoblot analysis showing CD68 and STING expression in paired normal mucosa and tumor samples from GC patients. GAPDH was used as a loading control.

