# Supplementary material for: Targeting the STING pathway in tumor-associated macrophages regulates innate immune sensing of gastric cancer cells
Source: Theranostics. 2020 Jan 1;10(2):498–515. doi: 10.7150/thno.37745 (PMC6929973; doi:10.7150/thno.37745)
Supplement: Supplementary file 1 — Supplementary figures and tables. [file thnov10p0498s1.pdf]

Table S1. Sequence of primers used in real-time PCR

	Forward Primer	Reverse Primer
Human IL1β	AGCTACGAATCTCCGACCAC	CGTTATCCCATGTGTCGAAGAA
Human IL6	ACTCACCTCTTCAGAACGAATTG	CCATCTTTGGAAGGTTCAGGTTG
Human iNOS	TTCAGTATCACAACCTCAGCAAG	TGGACCTGCAAGTTAAAATCCC
Human TNFα	CCTCTCTCTAATCAGCCCTCTG	GAGGACCTGGGAGTAGATGAG
Human Arg-1	GTGGAAACTTGCATGGACAAC	AATCCTGGCACATCGGGAATC
Human CXCL9	CCAGTAGTGAGAAAGGGTCGC	AGGGCTTGGGGCAAATTGTT
Human CXCL10	GTGGCATTCAAGGAGTACCTC	TGATGGCCTTCGATTCTGGATT
Human IFNG	TCGGTAACTGACTTGAATGTCCA	TCGCTTCCCTGTTTTAGCTGC
Human CCL2	CAGCCAGATGCAATCAATGCC	TGGAATCCTGAACCCACTTCT
Human β actin	CATGTACGTTGCTATCCAGGC	CTCCTTAATGTCACGCACGAT
Mouse IL1β	GAAATGCCACCTTTTGACAGTG	TGGATGCTCTCATCAGGACAG
Mouse IL6	CTGCAAGAGACTTCCATCCAG	AGTGGTATAGACAGGTCTGTTGG
Mouse iNOS	ACATCGACCCGTCCACAGTAT	CAGAGGGGTAGGCTTGTCTC
Mouse TNFα	CAGGCGGTGCCTATGTCTC	CGATCACCCCGAAGTTCAGTAG
Mouse Arg-1	CTCCAAGCCAAAGTCCTTAGAG	GGAGCTGTCATTAGGGACATCA
Mouse β actin	AAGGCCAACCGTGAAAAGAT	GTGGTACGACCAGAGGCATAC

Table S2. Array map of 40 human cytokines

A	POS1	POS2	CXCL13
B	Eotaxin	Eotaxin-2	G-CSF
C	GM-CSF	I-309	ICAM-1
D	IFNg	IL-1a	IL-1b
E	IL-1ra	IL-2	IL-4
F	IL-5	IL-6	IL-6R
G	IL-7	IL-8	IL-10
H	IL-11	IL-12p40	IL-12p70
I	IL-13	IL-15	IL-16
J	IL-17	MCP-1	MCSF
K	MIG	MIP-1a	MIP-1b
L	MIP-1d	PDGF-BB	RANTES
M	TIMP-1	TIMP-2	TNFa
N	TNFb	TNF RI	TNF RII

Supplemental Figures

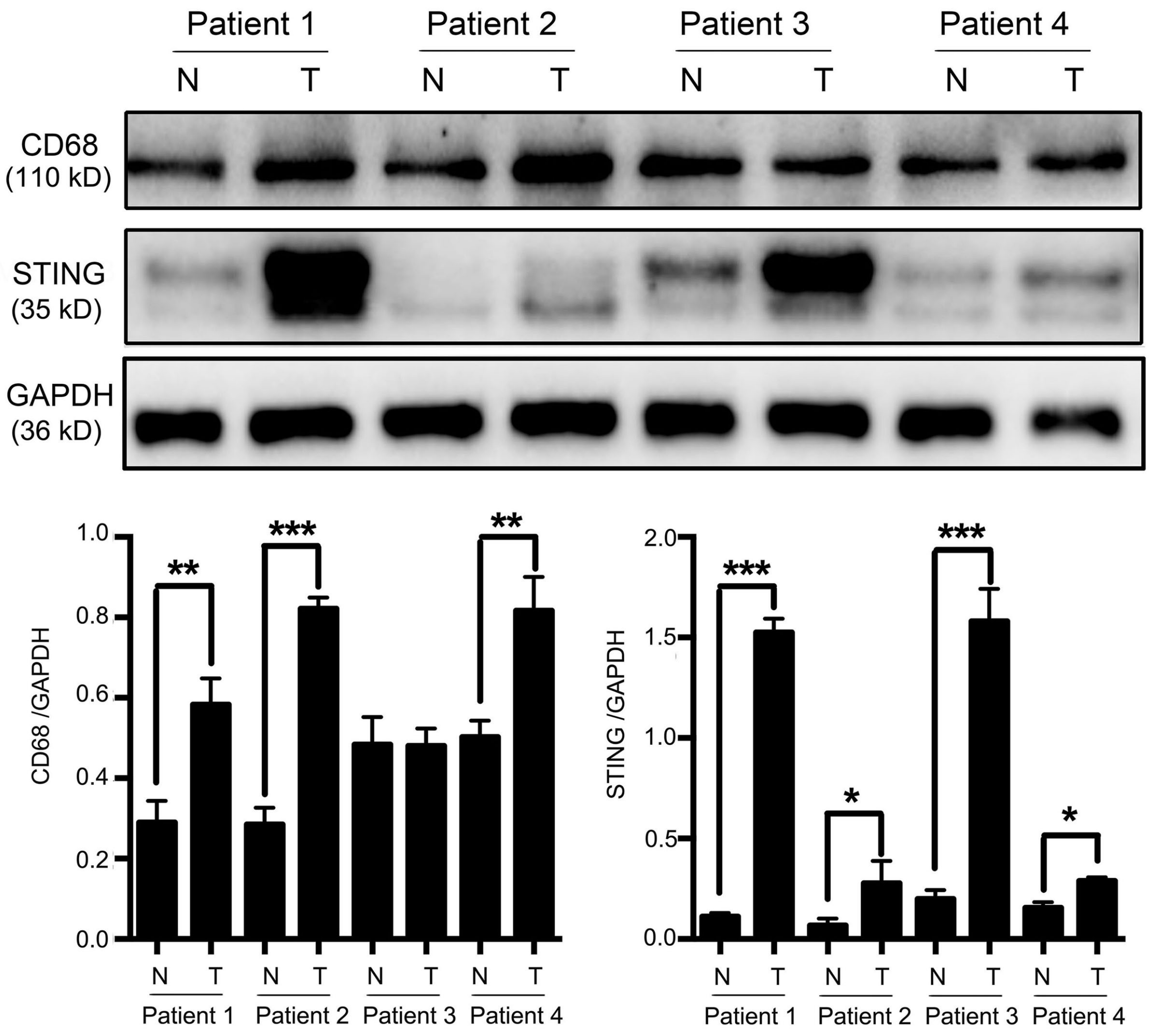

Figure S1. CD68 and STING expression in GC patient samples. Upper panel, immunoblot analysis showing CD68 and STING expression in paired normal mucosa and tumor samples from GC patients. GAPDH was used as a loading control. Lower panel, the ratios of CD68/GAPDH and STING/GAPDH were quantified, and statistical significance was analyzed by comparing to the normal mucosa. Data are presented as the mean±SD (n=3). *, $p < 0.05$; **, $p < 0.01$; ***, $p < 0.001$.

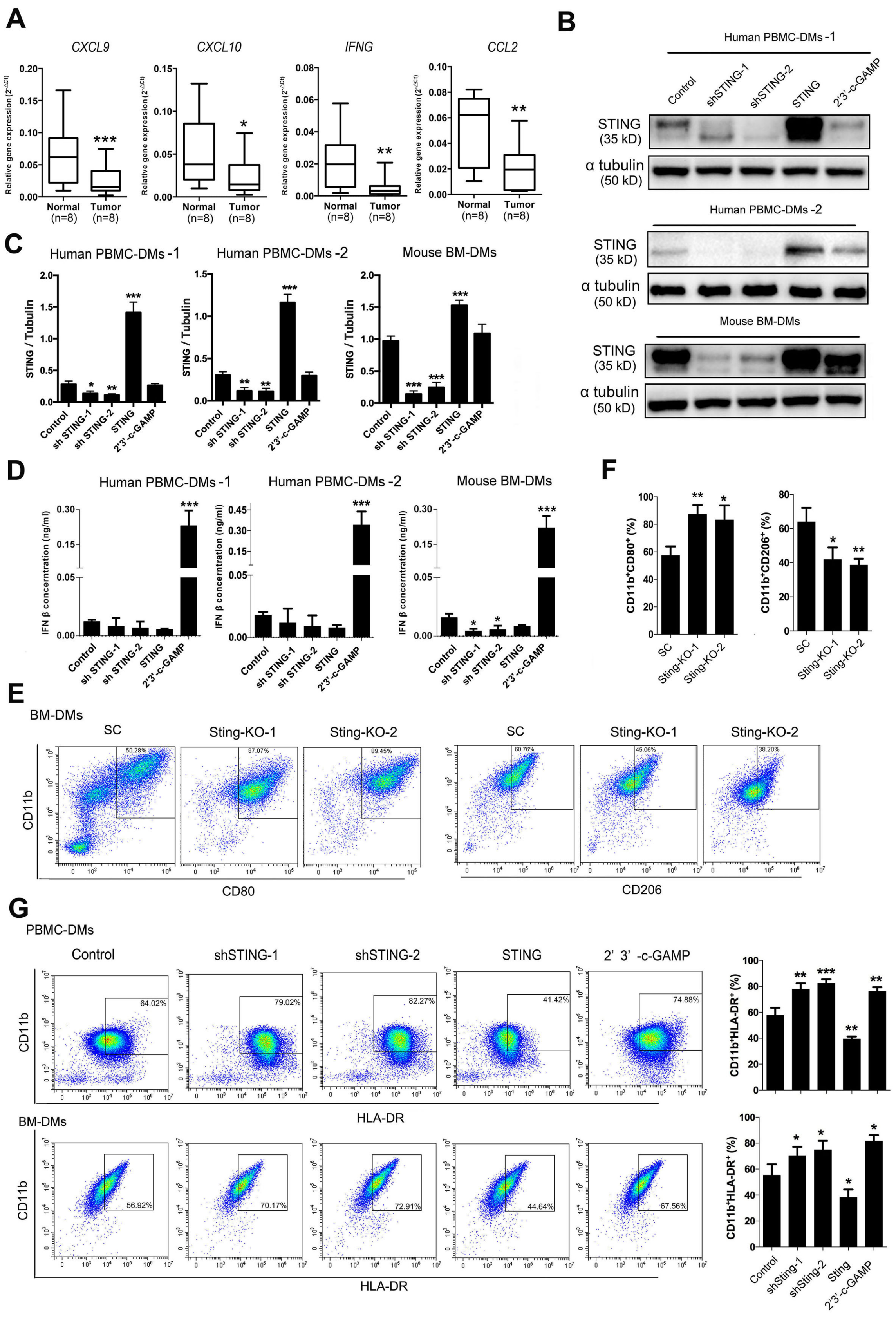

A
CXCL9
CXCL10
IFNG
CCL2
Relative gene expression (2^-ΔCt)
Normal (n=8)
Tumor (n=8)
B
Human PBMC-DMs -1
Control
shSTING-1
shSTING-2
STING
2'3'-c-GAMP
STING (35 kD)
α tubulin (50 kD)
Human PBMC-DMs -2
Mouse BM-DMs
C
Human PBMC-DMs -1
Human PBMC-DMs -2
Mouse BM-DMs
STING / Tubulin
D
IFN β concerntration (ng/ml)
F
CD11b+CD80+ (%)
CD11b+CD206+ (%)
SC
Sting-KO-1
Sting-KO-2
E
BM-DMs
CD11b
CD80
CD206
G
PBMC-DMs
HLA-DR
CD11b+HLA-DR+ (%)
shSting-1
shSting-2
Sting

Figure S2. Knocking-down and activating STING promotes both PBMC-DMs and BM-DMs differentiation into the pro-inflammatory subtype. (A) RT-PCR analysis of therapy-related cytokines in 8 pairs of adjacent normal mucosa and GC samples. ***, $p < 0.001$; **, $p < 0.01$; *, $p < 0.05$. **(B)** Immunoblot analysis showing STING expression in human peripheral blood-derived macrophages (PBMC-DMs) and mouse bone marrow-derived macrophages (BM-DMs) that 1) had been knocked-down STING (shSTING); 2) were ectopically expressing STING (STING); and 3) were treated with the STING activator 2'3'-c-GAMP. α-Tubulin was used as a loading control. The control represents a sample of three undistinguishable controls including scrambled sequence (SC) for shSTING, empty vector (EV) for STING overexpression, and PBS treatment for 2’3’-c-GAMP. The expression of STING in the three control samples was similar, so only one was shown as the control for clarity. **(C)** The ratio of STING/Vinculin was quantified, and statistical significance was analyzed by comparing to the control (that is the average of three controls). *, $p < 0.05$; **, $p < 0.01$; ***, $p < 0.001$. **(D)** IFN-β expression measured by ELISA in the supernatants of human PBMC-DM (left) or mouse BM-DM (right) cultures treated as indicated. *, $p < 0.05$; ***, $p < 0.001$. **(E, F)** Flow cytometric analysis of surface marker expression for pro-inflammatory ($CD11b^+/CD80^+$) and anti-inflammatory macrophages ($CD11b^+/CD163^+$) in mouse BM-DMs with Sting knock-out based on the CRISPR-Cas9 system; quantification in (F). *, $p < 0.05$; **, $p < 0.01$. SC represents control with scrambled gRNA transfectioin. **(G)** Left panel, representative flow cytometric plots of human PBMC-DMs treated as indicated; right panel, quantification. ***, $p < 0.001$; **, $p < 0.01$; *, $p < 0.05$. Data in B-G are presented as the mean±SD (n=3).

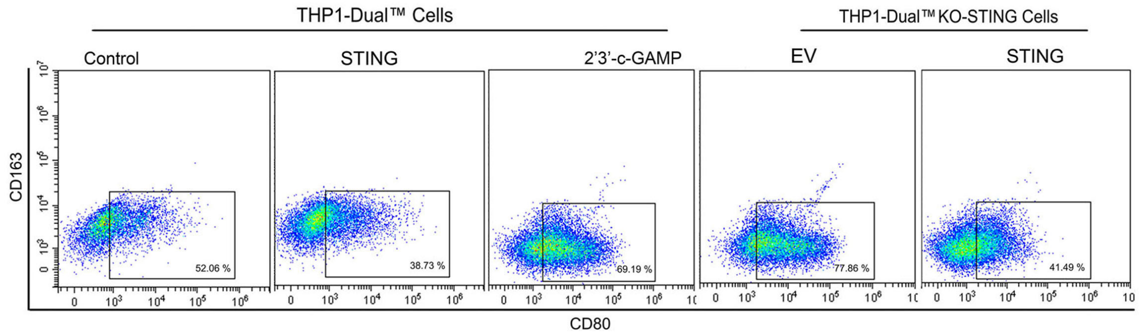

Figure S3. STING knock-out in THP1-derived macrophages promotes pro-inflammatory subtype differentiation. Representative flow cytometric plots of surface marker expression ($CD11b^{+}CD80^{+}$) in human THP1-derived macrophages treated as indicated. Control stands for the average of undistinguishable controls of scrambled sequence (SC; for shSTING), and PBS treatment (for 2'3'-c-GAMP treatment), empty vector (EV) was used as a control of STING overexpression.

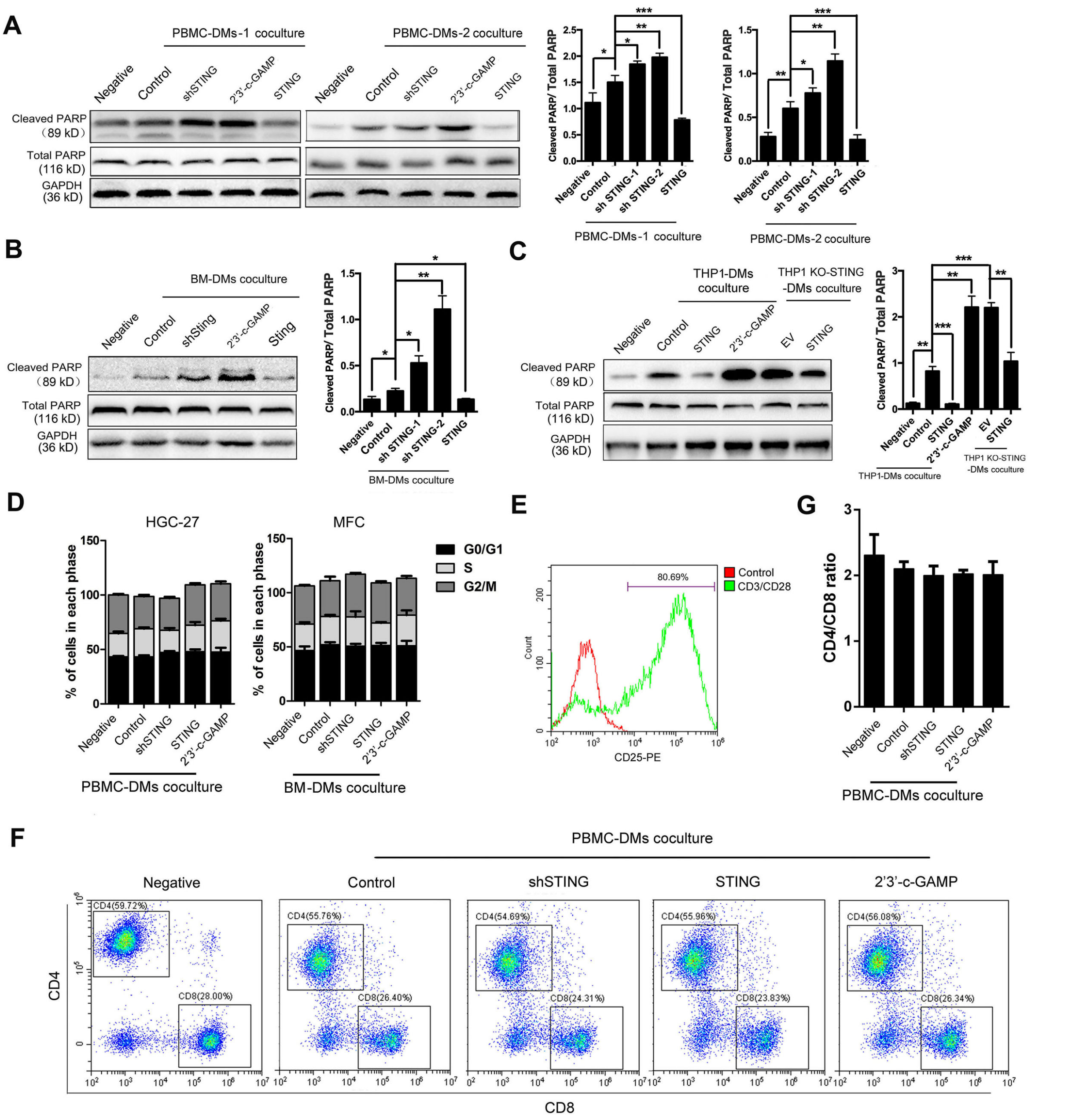

Figure S4. Macrophages with STING alteration had no effect on cell cycle arrest of GC cells or phenotype of T cells. (A-C) Left panels, immunoblot analysis of cleaved-PARP and total PARP expression in human PBMC-DMs (A), mouse BM-DMs (B) and THP1-DMs (C) treated as indicated. GAPDH was used as a loading control. Right panels, the ratios of Cleaved PARP/Total PARP were quantified; *, $p < 0.05$; **, $p < 0.01$; ***, $p < 0.001$. **(D)** Cell cycle analysis of human PBMC-DMs (left) and mouse BM-DMs (right) treated as indicated. **(E)** Isolated CD3$^+$ total T cells from human PBMCs were stimulated with ImmunoCult™ Human CD3/CD28 T Cell Activator and cultured in ImmunoCult™-XF T Cell Expansion Medium. The activation of viable CD3$^+$ T cells was assessed by CD25 expression using flow cytometry. **(F)** Representative flow cytometric plots of CD4/CD8 T cells in cocultures of human T cells with human PBMC-DMs treated as indicated; quantification of the CD4/CD8 ratio in **(G)**. (A, B, D, F, G) Control stands for a representative sample of undistinguishable controls of scrambled sequence (for shSTING), empty vector (for STING overexpression), and PBS treatment (for 2'3'-c-GAMP treatment). (C) Control stands for the average of undistinguishable controls of scrambled sequence (SC; for shSTING), and PBS

treatment (for 2’3’-c-GAMP treatment), empty vector (EV) was used as a control of STING overexpression. Data in A, B, C, D, G are presented as the mean±SD (n=3).

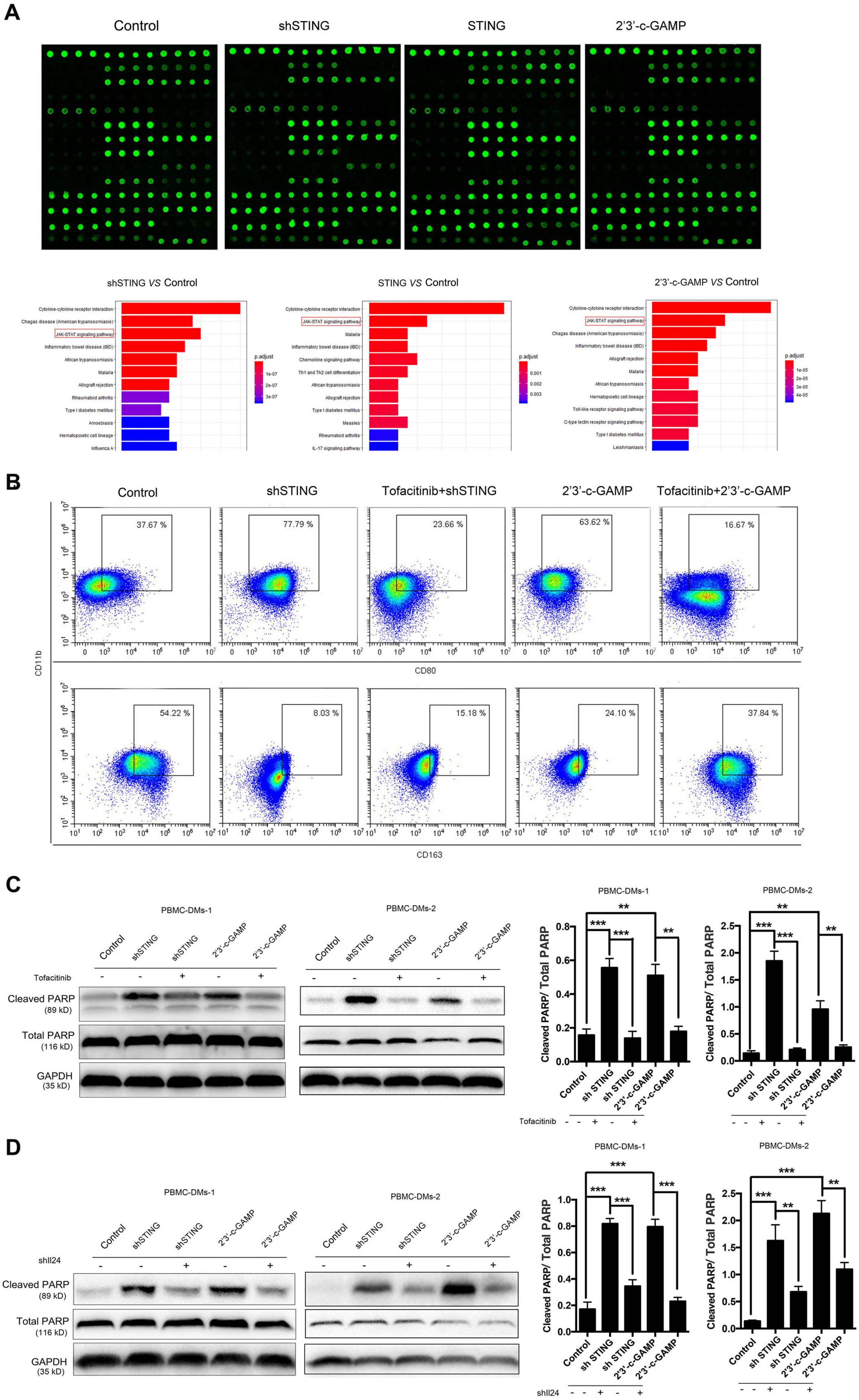
A
Control
shSTING
STING
2'3'-c-GAMP
shSTING VS Control
STING VS Control
2'3'-c-GAMP VS Control
p.adjust
B
Control
shSTING
Tofacitinib+shSTING
2'3'-c-GAMP
Tofacitinib+2'3'-c-GAMP
37.67 %
77.79 %
23.66 %
63.62 %
16.67 %
CD11b
CD80
54.22 %
8.03 %
15.18 %
24.10 %
37.84 %
CD163
C
PBMC-DMs-1
PBMC-DMs-2
Control
shSTING
2'3'-c-GAMP
Tofacitinib
Cleaved PARP
(89 kD)
Total PARP
(116 kD)
GAPDH
(35 kD)
Cleaved PARP/ Total PARP
sh STING
D
PBMC-DMs-1
PBMC-DMs-2
shIl24
Cleaved PARP
(89 kD)
Total PARP
(116 kD)
GAPDH
(35 kD)
Cleaved PARP/ Total PARP

Figure S5. The effects of STING knockdown and activation on macrophage differentiation are mediated through the JAK-STAT-IL24 pathway. (A) Upper panel, whole pictures of cytokine arrays in Figure 5A. Lower panel, enriched pathways of altered cytokines. Control stands for a representative sample of undistinguishable controls of scrambled sequence (for shSTING), empty vector (for STING overexpression), and PBS treatment (for 2'3'-c-GAMP treatment). **(B)** Representative flow cytometric plots of human PBMC-DMs in Figure 5C. **(C, D)** Left panels, immunoblot analysis of cleaved-PARP and total PARP expression in human PBMC-DMs treated as indicated. GAPDH was used as a loading control. Right panels, the ratios of Cleaved PARP/Total PARP were quantified; **, $p < 0.01$; ***, $p < 0.001$. Data in C and D are presented as the mean±SD (n=3) **(B-D)** Control stands for the average of undistinguishable controls of scrambled sequence (SC; for shSTING), and PBS treatment (for 2'3'-c-GAMP treatment), empty vector (EV) was used as a control of STING overexpression.

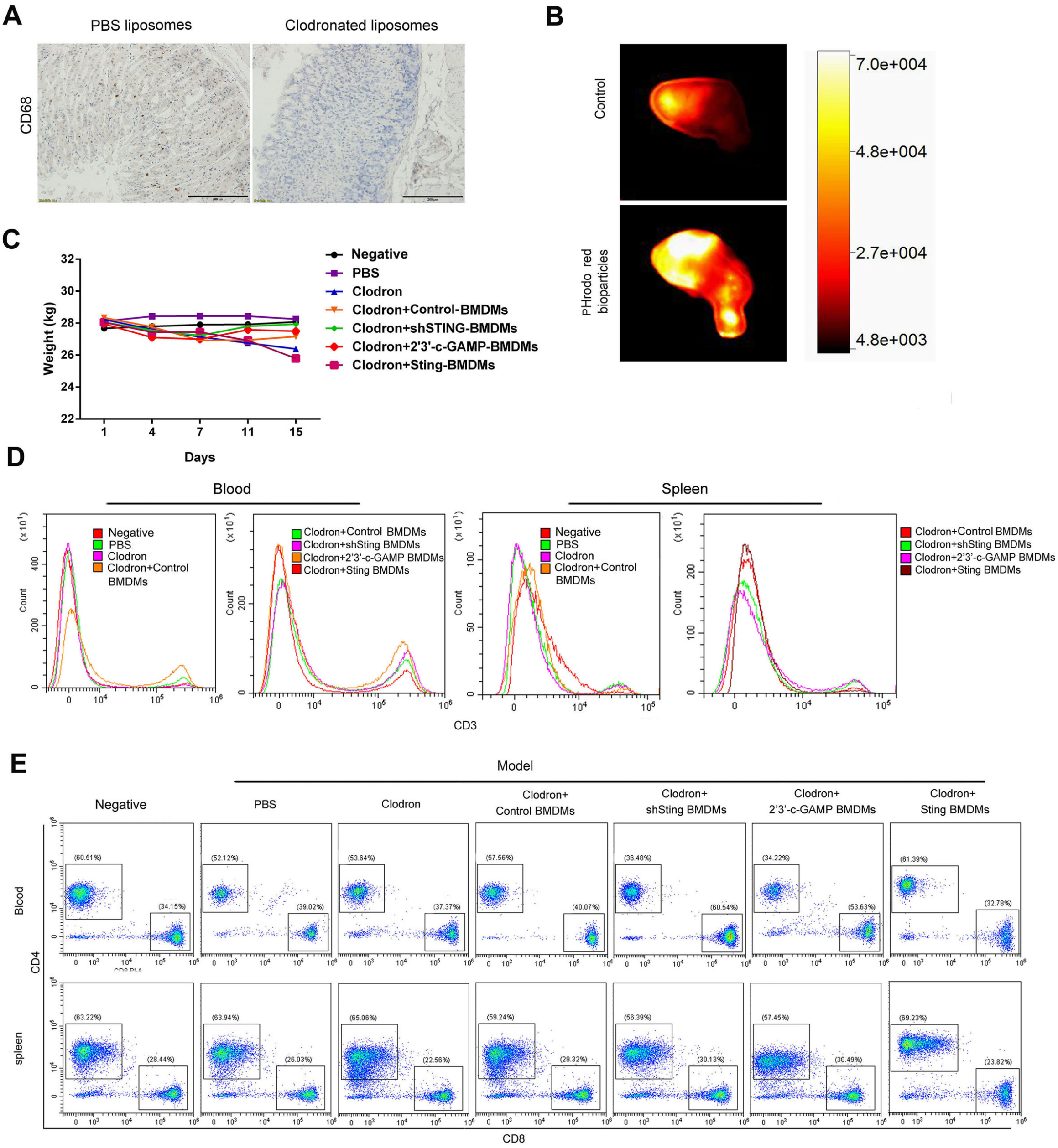

Figure S6. Macrophages with Sting knocking-down or activation have killing effects on cancer cells of spontaneous gastric tumors in mice. (A) Representative pictures of CD68 IHC staining in the spleens of mice treated as indicated(n=8). Scale bars represent 200 μm. **(B)** Body weight of mice with indicated treatments(n=8). **(C)** Immunofluorescence images indicating the engulfment of pHrodoTM red bioparticles by treated mice. Data are presented as the mean±SD (n=10). **(D)** Flow cytometric analysis of surface marker expression for total T cells (CD3^{+}) in the blood and spleens of mice treated as indicated. **(E)** Representative flow cytometric plots of mouse CD8^{+} and CD4^{+} T cells in monocytes (upper) and splenocytes (lower) in Figure 6E and F. (C-E) Control stands for a representative sample of undistinguishable controls of scrambled sequence (for shSting), empty vector (for Sting overexpression), and PBS treatment (for 2’3’-c-GAMP treatment).
